# From Data to Diagnosis: A Novel Deep Learning Model for Early and Accurate Diabetes Prediction

**DOI:** 10.3390/healthcare13172138

**Published:** 2025-08-27

**Authors:** Muhammad Mohsin Zafar, Zahoor Ali Khan, Nadeem Javaid, Muhammad Aslam, Nabil Alrajeh

**Affiliations:** 1 ComSens Lab, International Graduate School of Artificial Intelligence, National Yunlin University of Science and Technology, Douliu, Yunlin 64002, Taiwan; muhammadmohsinzafar@gmail.com (M.M.Z.); javaidn@yuntech.edu.tw (N.J.); 2 Faculty of Computer Information Science, Higher Colleges of Technology, Fujairah 4114, United Arab Emirates; 3 Department of Computer Science, Aberystwyth University, Aberystwyth SY23 3FL, UK; mua19@aber.ac.uk; 4 Department of Biomedical Technology, College of Applied Medical Sciences, King Saud University, Riyadh 11633, Saudi Arabia

**Keywords:** diabetes prediction, deep learning, adaptive synthetic oversampling, long short-term memory, multi-layer perceptron, inception network, confidence interval, k-fold cross validation, local interpretable model-agnostic explanations, Shapley additive explanations

## Abstract

**Background**: Diabetes remains a major global health challenge, contributing significantly to premature mortality due to its potential progression to organ failure if not diagnosed early. Traditional diagnostic approaches are subject to human error, highlighting the need for modern computational techniques in clinical decision support systems. Although these systems have successfully integrated deep learning (DL) models, they still encounter several challenges, such as a lack of intricate pattern learning, imbalanced datasets, and poor interpretability of predictions. **Methods**: To address these issues, the temporal inception perceptron network (TIPNet), a novel DL model, is designed to accurately predict diabetes by capturing complex feature relationships and temporal dynamics. An adaptive synthetic oversampling strategy is utilized to reduce severe class imbalance in an extensive diabetes health indicators dataset consisting of 253,680 instances and 22 features, providing a diverse and representative sample for model evaluation. The model’s performance and generalizability are assessed using a 10-fold cross-validation technique. To enhance interpretability, explainable artificial intelligence techniques are integrated, including local interpretable model-agnostic explanations and Shapley additive explanations, providing insights into the model’s decision-making process. **Results**: Experimental results demonstrate that TIPNet achieves improvement scores of 3.53% in accuracy, 3.49% in F1-score, 1.14% in recall, and 5.95% in the area under the receiver operating characteristic curve. **Conclusions**: These findings indicate that TIPNet is a promising tool for early diabetes prediction, offering accurate and interpretable results. The integration of advanced DL modeling with oversampling strategies and explainable AI techniques positions TIPNet as a valuable resource for clinical decision support, paving the way for its future application in healthcare settings.

## 1. Introduction

Healthcare is one of the most important aspects of society because it must be functional 24 h a day. Due to the rapidly growing population in urban areas, hospitals and other healthcare institutions must operate with high efficiency [1]. Manually monitored healthcare systems struggle to handle the flow of patient records. Healthcare operations include maintaining electronic health records (EHRs), diagnosing diseases, monitoring patient conditions, and performing surgeries. The level of regional development significantly influences the quality and accessibility of healthcare services. In some regions, common challenges include inefficient and insufficient medical practitioners, and lack of advanced medical infrastructure, automated tools for the diagnosis of diseases, and assistance with artificial intelligence (AI)-enabled devices in surgeries. In contrast, developed societies increasingly utilize wearable devices to track vital health parameters such as blood pressure, glucose levels, calorie intake, and sleep patterns.

However, EHRs are valuable for disease prediction, patients’ medical history analysis, and the study of disease spreading patterns, and factors causing diseases. EHRs are frequently employed in modern AI-enabled devices for early diagnosis and disease prediction. Balanced EHR data facilitates the efficient training of machine learning (ML) and deep learning (DL) models. However, privacy issues limit access to high-quality EHRs. Small and imbalanced EHRs datasets are unsuitable for the application of DL techniques.

Diabetes is one of the world’s major causes of death [2]. It affects practically every family, which makes it well-known throughout the world. Patients suffer from diabetes because of the insufficient production of insulin, which is produced by the pancreas. Diabetes can be effectively managed if diagnosed and treated promptly, but there is no long-term cure. Patients suffering from diabetes face problems, such as hunger, thirst, joint complications, low immunity, and weight loss. Diabetes can be predicted very early with the help of DL models, which help patients to avoid severe health conditions [3]. A quick response to a patient’s critical condition is essential in assisting doctors and patients. To reduce the chance of mortality from diabetes, scientists from all around the world are working together to develop innovative and effective medical health systems for early diagnosis through identification of the factors that are responsible for diabetes [4]. If diabetes is detected in its early stages, the chances of effective management will be higher compared to detection in later stages.

Despite considerable advancements in predictive modeling for diabetes, several critical challenges remain unaddressed. Many existing approaches are limited in their ability to simultaneously process static health indicators, such as demographic and lifestyle factors, alongside temporal patterns derived from physiological data. This lack of comprehensive feature integration results in suboptimal model performance. Additionally, most predictive models suffer from biased learning due to severe class imbalance, leading to inaccurate and unreliable predictions, especially in minority class instances. Another significant gap lies in the interpretability and explainability of model outcomes, which is essential for clinical acceptance and decision-making support. Without providing a clear understanding of which features influence predictions, such models lack transparency and hinder trust in automated healthcare systems. Furthermore, the generalizability of many models is often not rigorously validated, raising concerns about their robustness across diverse patient populations. These limitations highlight the need for a predictive framework that can effectively integrate heterogeneous health data, address data imbalance, offer interpretable outputs, and demonstrate consistent performance through systematic evaluation.

To address the inherent complexities in diabetes prediction, we propose a novel DL architecture called the temporal inception perceptron network (TIPNet). TIPNet is specifically designed to handle both static and sequential health indicators. A long short-term memory (LSTM) network is integrated to capture temporal dependencies within sequential data such as glucose levels, mental and physical health trends, and activity logs. LSTM is well-suited for modeling long-term dependencies and mitigating the vanishing gradient problem, which frequently affects standard recurrent architectures. In parallel, the inception network (InceptionNet) is employed to extract multiscale representations from static features such as *age*, *BMI*, and *cholesterol* levels. Its architecture allows simultaneous processing at different receptive fields, enabling the model to capture both fine- and coarse-grained patterns. To effectively fuse the outputs of these two diverse feature extractors, a multilayer perceptron (MLP) is used as the meta-classifier. The MLP aggregates and refines the extracted features through its dense hidden layers and nonlinear activation functions, leading to robust final predictions. This blending ensemble approach allows TIPNet to exploit the complementary strengths of LSTM, InceptionNet, and MLP, thereby overcoming key limitations of previous methods in terms of pattern diversity, feature scale, and temporal modeling. In addition, to handle the severe class imbalance in the dataset, adaptive synthetic (ADASYN) oversampling is applied to generate synthetic samples for harder-to-learn instances, improving the model’s generalization and reducing biased classification. The performance of TIPNet is rigorously validated using k-fold cross validation (K-FCV), ensuring the robustness and stability of the results across different data splits. Finally, to enhance model transparency and trustworthiness, explainable AI techniques, including Shapley additive explanations (SHAP) and local interpretable model-agnostic explanations (LIME), are employed to interpret and explain feature importance and provide human-understandable insights into the model’s predictions. Here is the sequence of the remaining paper. Related work, problem statement, proposed diabetes diagnosis model, results, and conclusion are presented in Section 2, Section 3, Section 4, Section 5, and Section 6, respectively. Abbreviations and symbols used in this study are listed in Abbreviations.

## 2. Related Work

The authors in [5] use the PIMA dataset containing information about the patients’ pregnancies, glucose levels, blood pressure, and age. Because of the imbalanced nature of the dataset, variable AE (VAE) is employed for the augmentation of data from the minority class, and sparse AE (SAE) is used for the feature augmentation because the original dataset contains only eight features. Convolutional neural network (CNN) and MLP classifiers are used to predict diabetic patients. It is noted from the results that support vector machine (SVM), decision tree (DT), random forest (RF), XGBoost, KNN, and MLP had higher accuracies on augmented or oversampled datasets as compared to the original dataset. Limitation: The CNN and MLP framework with VAE and SAE augmentation relies on synthetic data generation, which can lead to overfitting and does not account for temporal dependencies in the data. This limits its ability to model sequential patterns crucial for diabetes prediction. TIPNet Solution: As noted in Table 1, TIPNet naturally handles imbalanced data without oversampling and integrates LSTM for capturing temporal dependencies, while the InceptionNet module extracts diverse static features, leading to better generalization and improved prediction performance.

The authors in [6] conducted a comparative study of the most popular ML classifiers and an ontology-based ML classification. PIMA is a binary dataset used for training and evaluation. The authors use SWRL rules in conjunction with ML techniques for ontological classification. To evaluate the results of ML techniques, 10-FCV is also implemented. Limitation: The ontology-based ML classification with SWRL rules [6] depends on manually designed rules, which limits adaptability to new or unseen data. This manual dependency reduces scalability and the ability to learn complex, data-driven patterns. TIPNet Solution: As shown in Table 1, TIPNet learns patterns directly from data using deep feature extraction via InceptionNet and LSTM, eliminating the need for handcrafted rules and improving adaptability to diverse datasets.

A study is conducted in [7] to investigate the importance of environmental chemical exposure in predicting diabetes mellitus. A dataset of 8501 individuals is collected from the National Health and Nutrition Examination Survey from 2005 to 2016. The authors employed RF and least absolute shrinkage and selection operator (LASSO) regression in the discovery set for the optimal selection of the features with 10-FCV and built a diabetes prediction model. RF led to poor performance and overfitting, but LASSO performed better and had interpretability because it removed irrelevant features. Limitation: The RF and LASSO regression framework suffers from overfitting in RF and limited expressiveness in LASSO, which restricts the ability to capture complex non-linear and temporal interactions in the data. TIPNet Solution: As highlighted in Table 1, TIPNet is robust to irrelevant features through deep feature learning and effectively captures both non-linear relationships and temporal dependencies using its integrated InceptionNet and LSTM modules.

The authors in [8] performed a comparative study by using different ML techniques. The behavioral risk factor surveillance system (BRFSS) dataset is used. BRFSS contains 253,680 instances with 22 features. Data is highly imbalanced with only 14% of diabetic samples and 86% non-diabetic instances. For dataset balancing, the authors employ synthetic minority oversampling technique (SMOTE). Principal component analysis (PCA), a feature selection technique, is used due to its unsupervised nature. Five different ML classifiers, DT, RF, KNN, LR, and naive bayes (NB), are employed for the prediction performance comparison. Among the five ML classifiers, RF showed better performance. Limitation: The ML framework with PCA and SMOTE is limited by SMOTE’s tendency to oversample noisy instances and its lack of temporal modeling capability, which may lead to overfitting and reduced performance on sequential or complex healthcare data. TIPNet Solution: As presented in Table 1, TIPNet avoids synthetic oversampling by using ADASYN to focus on harder-to-learn samples and leverages LSTM for temporal modeling, while the InceptionNet module captures deep latent features for robust classification.

A hybrid framework of artificial neural network (ANN) and genetic algorithm (GA) is proposed in [9] for robust prediction of diabetes. The proposed framework uses a novel regularization technique to avoid a severe overfitting problem. Most of the time real-world datasets do exhibit some level of skewness. To mitigate this issue, the authors propose a novel normalization technique and claim that this normalization is robust to any level of skewness in the dataset. The most influential variables or features of the PIMA dataset are also identified, which are further used for the efficient classification of diabetic and non-diabetic samples with the help of the proposed hybrid model of ANN and GA. Limitation: The ANN and GA-based framework lacks temporal modeling capabilities and may converge to local minima, limiting its ability to capture long-term dependencies and complex feature interactions in medical data. TIPNet Solution: As indicated in Table 1, TIPNet integrates LSTM to effectively learn long-term temporal patterns and uses InceptionNet for diverse feature extraction, improving stability and generalization across heterogeneous datasets.

In [10], a cloud-based architecture of DL and ML approaches is built for the classification of patients into diabetic and non-diabetic groups. A fused ML approach is used for the final decision making. The binary dataset has a total of 520 instances with 17 features. The proposed fused model for diabetes prediction (FMDP) cloud-based framework first performs data acquisition and normalization in preprocessing, then for the classification of preprocessed data, employs SVM and ANN. Limitation: The cloud-based ANN and SVM fusion framework is limited by its small dataset size and restricted scalability, which may hinder performance and generalization on larger and more diverse healthcare datasets. TIPNet Solution: As shown in Table 1, TIPNet is scalable to large datasets and can operate effectively in both cloud and offline environments, leveraging deep feature extraction and temporal modeling for improved predictive accuracy.

The authors in [11] performed a comparative study on the PIMA binary dataset using multiple ML techniques. In the preprocessing step, the outliers are removed, and missing value imputation is performed using the mean technique. The original PID dataset contains 768 instances, but after dataset cleaning in the preprocessing step, it is reduced to 722 records with six features. Six ML techniques: KNN, NB, Extremely randomized tree (ERT), radial basis function, and MLP are used in this comparative study. Limitation: The comparative study using KNN, NB, ERT, RBF, and MLP relies on shallow learning models that lack hierarchical feature extraction capabilities, limiting their ability to capture complex, multi-scale patterns in the data. TIPNet Solution: As highlighted in Table 1, TIPNet employs InceptionNet for multiscale deep feature extraction and LSTM for temporal modeling, enabling better generalization and improved performance compared to shallow models.

In [12], the authors proposed an electronic diagnosis system based on the ML technique for predicting type 2 diabetes mellitus (T2DM). This study utilizes the PIMA dataset. Features having 0 values are imputed using the median technique. Three feature selection methods are used: PCA, KMC, and importance ranking. The SHAP summary plot is used to determine the importance of the features. Three different inputs are created: only imputation, with only three and five factors, and all three models are evaluated. Limitation: The ML-based diagnosis system with PCA, KMC, and SHAP is limited to modeling static patterns and lacks adaptability to complex, non-linear, and temporal relationships present in healthcare data. TIPNet Solution: As indicated in Table 1, TIPNet implicitly learns important features through DL without relying solely on manual feature selection and is adaptive to complex decision surfaces by combining multiscale static feature extraction with temporal modeling.

PyCaret is used to implement ML techniques in [13] for efficiently classifying diabetic and non-diabetic patients. The PID binary class dataset is used in this research work, and it has a total of 768 samples with 19 features. The authors performed exploratory data analysis to check the relationship between variables and later used it for feature engineering. Initially, a comparative experiment is held to check the performance of 14 ML techniques: LR, ridge classifier, linear discriminant analysis, RF, adaptive boosting, gradient boosting classifier (GBC), light gradient boosting, ETC, KNN, NB, dummy classifier, quadratic discriminant analysis, and SVM with linear kernel. Limitation: The PyCaret-based comparative study focuses solely on traditional ML techniques without integrating DL, resulting in poor temporal insight and limited ability to capture complex feature interactions in healthcare data. TIPNet Solution: As shown in Table 1, TIPNet combines deep and shallow feature learning by integrating LSTM for temporal dependencies and InceptionNet for diverse static feature extraction, eliminating the need for extensive manual tuning while improving predictive performance.

For the early prediction of T2DM, a study is carried out in [14], where the authors used a self-collected survey-based dataset and proposed a framework for the classification of diabetic samples. A total of 1552 samples with 11 attributes were collected under the supervision of medical experts like endocrinologists and nutritionists, from different regions of Jammu and Kashmir. Seven different ML techniques, KNN, LR, SVM, NB, DT, RF, and gradient boosting (GB), are implemented on this lifestyle indicators-based dataset. In the data preprocessing step, outliers and wrong values are removed, and null values are imputed using mean, median, and mode techniques. A standard scaler is also implemented to reshape the values of features in the range of −1 and 1. Limitation: The survey-based ML framework is constrained by a limited feature space and the use of traditional models, which restrict its ability to capture complex relationships between lifestyle and clinical indicators for T2DM prediction. TIPNet Solution: As highlighted in Table 1, TIPNet simultaneously adapts to lifestyle and clinical features through its InceptionNet module for multiscale static feature extraction and LSTM for temporal dependency modeling, ensuring robust performance under diverse conditions.

The authors of [15] presented a new hybrid framework for diabetes and glucose level prediction that is based on DL and meta-heuristics. The moth flame-based crow search algorithm (MF-CSA) was used by the authors to address the multi-objective optimization problem. This study utilizes the PIMA dataset. Firstly, features are extracted using a 2-layer CNN, and several neurons in each layer are optimized using the proposed MF-CSA technique; then, based on these extracted features, a modified fuzzy classifier is used along with the optimized membership function for diabetes detection. Features extracted from the optimized CNN are fed to the recurrent neural network (RNN) to predict glucose level while minimizing the RMSE and mean absolute squared error (MASE). The MF-CSA optimization technique is used to optimize diabetes detection and glucose level prediction. Limitation: The hybrid CNN–fuzzy classifier–RNN framework with MF-CSA optimization suffers from high model complexity and the use of large convolutional filters, which can cause significant information loss during feature extraction. TIPNet Solution: As shown in Table 1, TIPNet employs lightweight convolutional filters in the InceptionNet module to preserve fine-grained details and combines them with LSTM for efficient modeling of both static and sequential features, reducing complexity while maintaining high predictive accuracy.

An ML-based system model is proposed in [16] for the early prediction of T2DM. For this study, a survey-based binary dataset, the Indian demographic and health survey (IDHS) 2016, is used. IDHS contains 676,677 samples and 13 features, having only 1.38% diabetic instances. The authors employed Kernel entropy component analysis (KECA) for dimension reduction and SMOTE for dataset balancing. Several different ML classifiers are deployed for prediction performance comparison, including LR, RF, SVM, Gaussian NB, linear discriminant analysis (LDA), KNN, DT, and extreme gradient boosting. Three experiments are conducted: without KECA and SMOTE, with KECA, and with both KECA and SMOTE. Limitation: The KECA and SMOTE-based ML framework is prone to overfitting due to synthetic oversampling by SMOTE and lacks deep representation learning capabilities, limiting its ability to optimally extract discriminative features from highly imbalanced datasets. TIPNet Solution: As indicated in Table 1, TIPNet naturally handles skewed data without relying on synthetic oversampling and employs InceptionNet for multiscale feature extraction along with LSTM for temporal modeling, enabling robust and adaptive predictions.

The authors in [17] propose an ensemble model for predicting T2DM using a survey-based self-collected dataset of lifestyle indicators. The dataset is collected from the different hospitals in Jammu and Kashmir from 2019 to 2021. It comprises 1939 samples and 11 features. In the preprocessing step, missing values are imputed using the average method, and outliers are detected using a boxplot, which uses the interquartile range method; detected outliers are replaced with feasible values. The SMOTE oversampling technique is employed for balancing. The authors proposed three ensemble techniques: bagging, boosting, and voting for the prediction of T2DM. Bagging used RF, bagged tree, and extra tree, boosting implemented AdaBoost and SGB, and voting used LR, ST, and SVM. The bagged decision tree outperformed all other ensemble learning models. Limitation: The ensemble learning framework with SMOTE oversampling increases model complexity and is prone to overfitting due to synthetic data generation, while also lacking deep temporal modeling capabilities for healthcare time-series data. TIPNet Solution: As presented in Table 1, TIPNet reduces the need for complex ensembling by internally fusing multiscale features via InceptionNet and modeling temporal dependencies through LSTM, thereby avoiding synthetic oversampling and improving time-aware predictions.

Blood glucose level prediction models have recently been developed and incorporated into the internet of medical things (IoMT) for real-time blood glucose monitoring. However, the main problem is the limited resources of IoMT. A framework for attention-based evidential RNN is developed in [19] for the real-time prediction of blood glucose levels. The authors also develop a Bluetooth-based hardware device that collects the real-time blood glucose level every 5 min, and edge computing is incorporated in the proposed framework for cost-effective real-time blood glucose prediction. Three different datasets, OhioT1DM, ABC4D, and ARISES, are used in model evaluation. Limitation: The attention-based evidential RNN framework achieves sequence modeling but has high training time and is sensitive to noise, which may reduce efficiency and robustness in real-time healthcare applications with resource constraints. TIPNet Solution: As noted in Table 1, TIPNet employs LSTM for efficient sequence learning with low latency and integrates InceptionNet for robust multiscale feature extraction, improving resilience to noise while maintaining computational efficiency.

In [18], the authors proposed DL and an attention-based fast-adaptive and confident neural network (FCNN) framework for blood glucose prediction in T1DM. This study utilizes three distinct multivariate time series datasets: OhioT1DM, ABC4D, and ARISES. To develop an RNN-based model for extracting feature maps from multivariate time series, the authors implemented three bidirectional GRU (BiGRU) layers, and a many-to-one attention layer is also employed on the output from BiGRU, which helps to focus on the essential parts of hidden representations. Limitation: The BiGRU with attention-based FCNN framework effectively captures important sequence information but suffers from high training time and sensitivity to noise, making it less suitable for scenarios requiring fast and robust predictions. TIPNet Solution: As highlighted in Table 1, TIPNet leverages LSTM for efficient sequence modeling with lower computational overhead and combines it with InceptionNet for robust multiscale feature extraction, improving both speed and resilience to noisy data.

## 3. Problem Statement

In [16,17,20], SMOTE is used for dataset balancing, which does not consider the decision boundary and performs oversampling for all the minority class instances, which leads to overfitting. The authors in [21] proposed a visual geometry group-16 (VGG16)-based model for diabetes classification. However, VGG16 has a very high number of parameters (144 million), which leads to slow training and high computational costs. The gradient problem has become insignificant, necessitating a broader range of feature selection techniques. In [15], the authors employed a hybrid model where a CNN is used for feature extraction. The proposed CNN uses 11×11 filters in the first convolution layer to extract features. However, a lot of information is lost due to the large filter size. BiGRU was used in [15,18,19] for diabetes prediction. However, GRU could perform better when there are complex features to extract and the data presents long-term dependencies. BiGRU is a complex model with a very high execution time and cannot learn long sequences. The authors in [22] employed a dense neural network (DNN) to predict diabetes. However, it needs help handling complex patterns prone to gradient vanishing and overfitting problems.

The comparison of different models with the proposed TIPNet is mentioned in Table 1. A mapping of identified limitations in the previous research works to the proposed solutions in this study is presented in Table 2. In this context, *L* denotes the identified limitation, while *S* represents the corresponding solution.

## 4. Proposed Diabetes Prediction System Model

**Figure 1:**
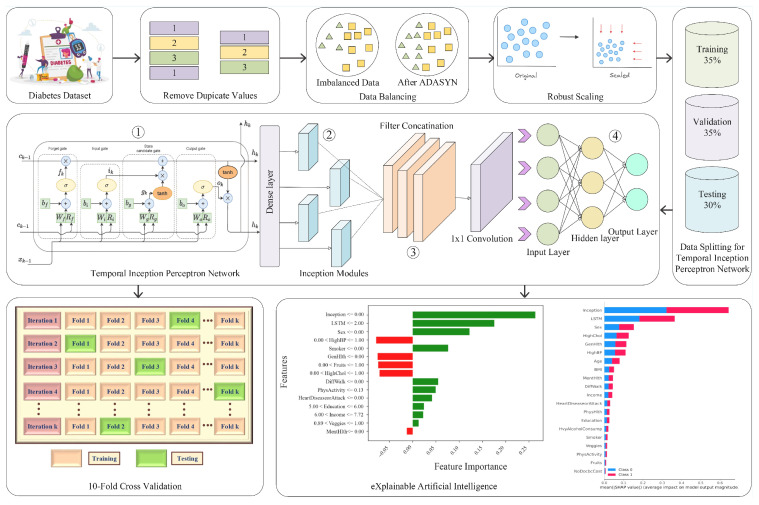
Proposed system model diagram for early and accurate diabetes prediction.

This section provides an overview of the proposed model designed for the accurate and early prediction of diabetes. The proposed system model starts with removing duplicate rows, then ADASYN is used to address the inherent class imbalance problem, ensuring fair representation of both diabetic and healthy cases in the training data. Lastly, a novel ensemble deep model, TIPNet, is proposed for the classification of diabetic and healthy cases. The section’s detailed illustration is shown in Figure 1, and the methodology is summarized in Table 3.

### 4.1. Description of Behavioral Risk Factor Surveillance System Dataset

This study uses the Diabetes Health Indicators Dataset. The data comes from the BRFSS, which is an annual health survey conducted by the centers for disease control and prevention (CDC) since 1984 to gather health information from more than 4 million adults [23]. The data for this study is extracted from the BRFSS2015 dataset available at the CDC with 22 features, including demographic characteristics (age, gender, race), lifestyle factors (physical activity, smoking habits), and medical history (diabetes, hypertension, cholesterol levels). This dataset has 253,680 respondents, which presents a clear picture of the health situations in the US population. Table 4 describes the diabetes health indicators dataset’s features and values. The data is free of outliers, missing values, and distortions. However, the dataset has a high class imbalance, with 86% of the instances in class 0 (non-diabetic) and the remaining 14% in class 1 (diabetic). Predictive modeling is challenged by this imbalance since models are possibly biased in favor of the majority class, which could reduce the precision of diabetes prediction. As it includes a wide range of health indicators associated with diabetes and other chronic conditions, the dataset can be used to develop and evaluate ML and DL models aimed at early diabetes detection.

#### 4.1.1. Removing Duplicate Rows

To ensure the integrity of the training and testing process, the dataset should not contain duplicate records, as models must be trained on unique instances to learn meaningful patterns. The presence of duplicate records does not contribute to the learning process and can lead to overfitting, where the model memorizes patterns instead of generalizing effectively. To address this issue, we examined our dataset for redundant records and identified 24,206 duplicate instances. These records are removed using the *drop_duplicates* function in Python, ensuring that each instance in the dataset remains unique. Mathematically, the dataset *D* after removing duplicates can be represented as:

(1) $$D^{\prime }=D-D_{\mathrm{dup}}$$

where *D* is the original dataset, $D_{\mathrm{dup}}$ represents the set of duplicate records, and $D^{\prime }$ is the cleaned dataset containing only unique instances. This preprocessing step enhances the reliability of the model by ensuring it learns from diverse and distinct data points.

#### 4.1.2. Robust Scaler

To ensure numerical stability and mitigate the influence of outliers, we applied the robust scaler to normalize continuous features such as *BMI*, *MentHlth*, and *PhysHlth*. Unlike standard normalization techniques that rely on the mean and standard deviation, the Robust Scaler transforms data using the median and interquartile range, making it less sensitive to extreme values. The transformation is mathematically defined as:

(2) $$X_{\mathrm{scaled}}=\frac{X-\mathrm{Median}(X)}{\mathrm{IQR}(X)}$$

where *X* represents the original feature value, Median(X) denotes the median of the feature, and IQR(X) is the interquartile range, computed as the difference between the 75th and 25th percentiles. This normalization technique ensures that the model learns from meaningful patterns in the data without being skewed by extreme values.

### 4.2. Description of DiaHealth Dataset

Similarly, the DiaHealth dataset, named DiaHealth: A Bangladeshi Dataset for Type 2 Diabetes Prediction, is a comprehensive and high-quality resource comprising medical and demographic records of 5437 patients [24]. It is specifically designed to support the development and evaluation of ML models aimed at the detection, prediction, and management of Type 2 Diabetes. Table 5 describes the DiaHealth dataset’s features and values. This dataset includes 14 independent features that capture a wide range of patient information relevant to diabetes diagnosis. These features are *age*, *gender*, *pulse rate*, *systolic blood pressure*, *diastolic blood pressure*, *glucose level*, *body mass index (BMI)*, *smoking history*, *physical activity*, *hypertension*, *cardiovascular disease*, *family history of diabetes*, *cholesterol level*, and *dietary habits*. Each instance in the dataset is labeled with a binary target variable indicating whether the patient is diagnosed with diabetes (positive class) or not (negative class). The dataset captures clinically relevant variables that are commonly used in real-world diagnostic settings, making it suitable for evaluating model generalizability across populations beyond the diabetes health indicators dataset. No preprocessing was applied to the dataset because it was already clean, with no missing or duplicate values.

### 4.3. Balancing the Highly Imbalanced Diabetes Health Indicators Dataset with Adaptive Synthetic Oversampling

Most real-world datasets are inherently imbalanced, where one class significantly outnumbers the others due to the rarity of certain events. For instance, a person’s chance of being sick is much lower than that of healthy people. A DL model trained on an imbalanced dataset will mostly classify new instances as majority class instances; it might indicate a misclassification. Models trained on the imbalanced dataset do not generalize efficiently. Therefore, it is essential to balance the dataset before training to ensure fair and accurate model performance.

The dataset used in this study is highly imbalanced, with only 14% instances of positive (1) class and 86% negative (0) class instances. Positive class instances are individuals with diabetes, and negative class samples are of healthy individuals. The minority class instances are synthetically oversampled using the ADASYN oversampling technique. We employed ADASYN because of its ability to learn adaptively and move decision boundaries towards harder-to-learn samples [25].

ADASYN is an oversampling technique, a variant of SMOTE [26]. It creates synthetic minority class samples by using existing sample patterns rather than just replicating the minority class samples. When it comes to synthetic sampling, other techniques randomly select the minority samples, while ADASYN selects the harder-to-learn samples. For each sample that is more difficult to learn, density is calculated to determine how many synthetic samples should be created near it. ADASYN’s adaptive nature allows it to generate more samples for harder-to-learn minority samples, which reduces the likelihood of biased predictions and shifts the decision boundary toward these samples. ADASYN operates in the following steps. Step 1 computes the imbalance ratio using Equation (3):

(3) $$d=\frac{m_{s}}{m_{l}}$$

where $m_{s}$ represents minority class samples and $m_{l}$ represents majority class samples. Step 2 computes the number of minority class samples to generate with the help of Equation (4):

(4) $$G=(m_{l}-m_{s})\times \beta$$

where β is the balance percentage in the range of [0,1]. In Step 3, ADASYN finds *K* Nearest Neighbors for each minority class sample $x_{i}$ using Equation (5).

(5) $$r_{i}=\frac{majority}{K}$$

The ratio $r_{i}$ represents the proportion of majority samples to total samples in the vicinity of a minority class sample $x_{i}$. Step 4 performs normalization on $r_{i}$ values for density calculation using Equation (6).

(6) $${\hat{r}}_{i}=\frac{r_{i}}{\sum r_{i}}$$

Step 5 calculates synthetic samples for $x_{i}$ with Equation (7):

(7) $$g_{i}={\hat{r}}_{i}\times G$$

where *G* represents the total synthetic samples to generate. Step 6 generates synthetic samples for each $x_{i}$ with the help of Equation (8).

(8) $$s_{i}=x_{i}+\left(x_{zi}-x_{i}\right)\times \lambda$$

In this context, $x_{zi}$ represents the KNN of $x_{i}$, and λ is a random number that falls within the range of [0,1]. ADASYN is crucial for enhancing predictive modeling in the context of diabetes and healthcare since it reduces class imbalance. Their adaptive generation of synthetic minority samples ensures that models learn from underrepresented diabetic cases, improving prediction accuracy and generalization. By shifting decision boundaries toward more difficult-to-classify data, ADASYN ultimately contributes to more equal healthcare outcomes and early diabetes detection by reducing bias in model learning.

### 4.4. Data Splitting

In our study, we performed a two-step data splitting strategy to train, validate, and test the proposed model. Initially, the balanced dataset is split into training and testing sets using a 70:30 ratio. Subsequently, the training set is further divided equally to create a validation set, i.e., 50% of the training set. This results in a final split of 35% training, 35% validation, and 30% testing data. The splits are performed using train_test_split with a fixed random seed (random_state = 42) to ensure reproducibility.

### 4.5. Newly Proposed Temporal Inception Perceptron Network for Diabetes Prediction

In this study, we present a novel architecture called TIPNet that addresses the challenges associated with diabetes prediction by employing a blended ensemble strategy. TIPNet is designed to handle both sequential and static medical data by integrating LSTM networks and InceptionNet in the base layer, while an MLP serves as a meta-classifier. This layered design enables TIPNet to capture temporal trends, extract multi-scale static features, and learn complex nonlinear decision boundaries, making it highly effective for heterogeneous medical datasets. Following preprocessing, the dataset is divided into training, validation, and testing partitions. The base models, LSTM and InceptionNet, are trained on the training set and validated on the validation set. Predictions generated by these base models are then used as inputs to train the MLP meta-classifier. The final performance of the ensemble is assessed on the independent test set [27]. To optimize prediction accuracy and minimize overfitting, TIPNet is trained by minimizing a generalized binary cross-entropy loss function. This loss function accounts not only for classification performance but also includes an $\ell_{2}$ regularization term to penalize model complexity:

(9) $$L\left(\theta \right)=-\frac{1}{N}\sum_{i=1}^{N}\left[y_{i}\log\left({\hat{y}}_{i}\right)+\left(1-y_{i}\right)\log\left(1-{\hat{y}}_{i}\right)\right]+\lambda {∥\theta ∥}_{2}^{2}$$

where *N* denotes the number of training samples, $y_{i}$ the ground-truth label, and ${\hat{y}}_{i}$ the predicted probability for sample *i*. The parameter θ includes all trainable weights and biases in the model, while λ regulates the trade-off between classification accuracy and model complexity. The LSTM component of TIPNet, as shown in Figure 1, is responsible for capturing sequential dependencies within features such as glucose levels, physical activity logs, and other time-series indicators. LSTMs are particularly advantageous due to their ability to preserve long-term dependencies and alleviate the vanishing gradient problem. The internal cell and hidden states are updated as follows:

(10) $$\begin{array}{cc} C_{t} & =\sigma \left(W_{f}h_{t-1}+U_{f}x_{t}+b_{f}\right)⊙C_{t-1}\\ \; & +\sigma \left(W_{i}h_{t-1}+U_{i}x_{t}+b_{i}\right)⊙\tanh\left(W_{c}h_{t-1}+U_{c}x_{t}+b_{c}\right) \end{array}$$

(11) $$h_{t}=\sigma \left(W_{o}h_{t-1}+U_{o}x_{t}+b_{o}\right)⊙\tanh\left(C_{t}\right)$$

In these equations, $x_{t}$ denotes the input at time step *t*, $h_{t}$ the hidden state, and $C_{t}$ the cell state. The matrices *W*, *U*, and biases *b* correspond to the forget, input, and output gates, and σ is the sigmoid activation function. These updates enable TIPNet to selectively remember and forget information over time, which is crucial for modeling the temporal progression of diabetes indicators. For static features such as *age*, *BMI*, and *cholesterol levels*, TIPNet leverages an InceptionNet module as illustrated in Figure 1 to perform multiscale convolution. This allows the model to extract patterns at various granularities. The convolutional operation at each path is given by:

(12) $$O_{i}=\sum_{k=1}^{K}\phi_{k}*X+b_{k}$$

where *X* is the input feature map, $\phi_{k}$ is the kernel for the *k*-th path, and $b_{k}$ is the bias term. The final static feature representation is obtained by concatenating the outputs from all paths:

(13) $$O=Concat(O_{1},O_{2},\ldots ,O_{K})$$

To further elaborate, the inception module applies convolutions of varying filter sizes 1×1, 3×3, and 5×5 alongside 3×3 max-pooling, which is summarized as:

(14) $$\begin{array}{c} O=Concat({Conv}_{1\times 1}\left(X\right),\\ \;\;\;\;\;\;\;\;\;{Conv}_{3\times 3}\left({Conv}_{1\times 1}\left(X\right)\right),\\ \;\;\;\;\;\;\;\;\;{Conv}_{5\times 5}\left({Conv}_{1\times 1}\left(X\right)\right),\\ \;\;\;\;\;\;\;\;{MaxPool}_{3\times 3}\left(X\right)) \end{array}$$

This multi-path setup enables the model to simultaneously detect fine-grained and broad spatial patterns within static data, which is particularly useful for complex clinical datasets. The features from the LSTM and InceptionNet modules are then fused to form a comprehensive representation that incorporates both temporal and static characteristics, as given in Figure 1. The fusion process is defined by:

(15) $$z=tanh(W_{c}\left[h_{T};O\right]+b_{c})$$

where $h_{T}$ is the last hidden state from the LSTM, *O* is the output from the InceptionNet, and $W_{c},b_{c}$ are weights and biases for the fusion layer. The concatenated vector *z* is then passed through the MLP meta-classifier, as shown in Figure 1, which consists of multiple fully connected layers:

(16) $$z^{\left(l\right)}=\sigma \left(W^{\left(l\right)}z^{\left(l-1\right)}+b^{\left(l\right)}\right),\;y=sigmoid\left(W_{out}z^{\left(L\right)}+b_{out}\right)$$

In this formulation, $z^{\left(l\right)}$ denotes the activation at the *l*-th hidden layer, with σ representing the *rectified linear unit (ReLU)* activation function. The final layer applies a sigmoid activation to generate the probability of diabetes. To prevent overfitting, TIPNet applies dropout regularization within the MLP during training:

(17) $$z_{dropout}^{\left(l\right)}=Dropout\left(z^{\left(l\right)},p\right)$$

where *p* is the dropout rate, indicating the probability of deactivating each neuron. Dropout increases generalization by reducing reliance on specific neurons. Additionally, batch normalization is used in the InceptionNet module to stabilize and accelerate training by normalizing intermediate activations. The TIPNet model is especially adept at modeling diverse data types. Its LSTM module captures temporal relationships such as fluctuations in glucose levels over time, while the InceptionNet module focuses on learning multiscale patterns among static features. For example, Equation (18) provides an alternate view of the LSTM gate calculations:

(18) $$f_{t}=\sigma \left(W_{f}\cdot \left[h_{t-1},x_{t}\right]+b_{f}\right),\;i_{t}=\sigma \left(W_{i}\cdot \left[h_{t-1},x_{t}\right]+b_{i}\right)$$

These gate activations control the flow of information through time, preserving meaningful temporal signals. The outputs of both modules are intelligently combined, allowing the MLP to leverage complementary information for final classification. This dual-path processing, temporal and static, enables TIPNet to achieve a more holistic and accurate prediction of diabetes. Ultimately, TIPNet’s architecture addresses both the representation and decision-making aspects of diabetes prediction. Its design ensures the model is both expressive and robust, integrating time-dependent and time-independent features through an ensemble strategy that leverages specialized submodules for different types of data. This synergy across modules significantly boosts predictive reliability and generalization on complex real-world medical datasets.

TIPNet implementation for diabetes prediction is described in Algorithm 1. First, the base models are built. Line 1 sets up the LSTM model to handle sequential data such as glucose trends. The six fully connected dense layers (Lines 3–7) and *n* hidden units (Line 2) each use *ReLU* activation for non-linearity. Then, to enable binary classification, a *sigmoid* activation function is defined for the output layer (Line 7). Line 8 uses the training data ($X_{train},Y_{train}$) to train the LSTM model, and Lines 9–10 produce predictions for the test and validation sets. In lines 12–28, the InceptionNet model is optimized for static features like age and BMI. Line 13 of the model architecture starts with input preprocessing. To capture features of different granularities, the model architecture uses multiscale convolutional filters such as 1×1 (Line 14), 3×3 (Lines 15–16), and 5×5 convolutions (Lines 17–18), in addition to 3×3 max pooling (Line 19). To avoid overfitting, the extracted feature maps are concatenated (Line 21), then run through a dense layer (Line 23) with dropout regularization (Line 24). The InceptionNet model is used to generate predictions for the validation sets (Lines 27–28).

The original validation data is combined with predictions from the LSTM and InceptionNet models in the ensemble phase (Lines 30–31). For the final diabetes prediction, these concatenated features are then passed to the meta-classifier, an MLP defined in Lines 32–39 that consists of four dense layers, each with *ReLU* activation, and a sigmoid output layer. Using the complementary strengths of the LSTM and InceptionNet base models, this pipeline combines static and temporal data to increase prediction accuracy.
**Algorithm 1**: Proposed Temporal Inception Perceptron Network (TIPNet) for diabetes prediction.
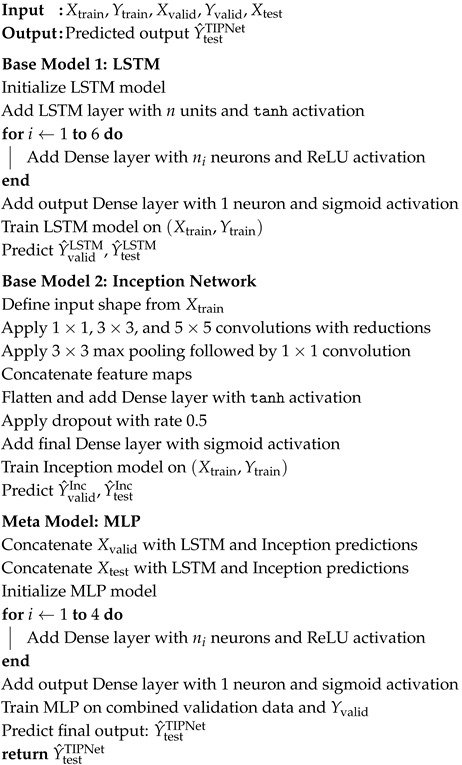


## 5. Simulations and Results

We have explained the proposed methodology in the previous section and claimed that the proposed system model will tackle the problems identified in the problem statement. To support the claimed results, we implemented our proposed system model and discuss them in this section. Then, 10-FCV is used to validate the results of the TIPNet deep model. SHAP and LIME are implemented to gain insight into the prediction of the TIPNet model. And finally, a thorough ablation study is conducted. The evaluation metrics used to validate the results of the proposed model are accuracy, recall, precision, F1-score, and area under the receiver operating characteristic (AUC-ROC). The formulas for these evaluation metrics are listed in Table 6. We initially reviewed the confusion matrix for a better understanding of the evaluation process. A confusion matrix is a performance evaluation parameter that divides the predictions made by the DL model into four categories, which are true positive (TP), true negative (TN), FP, and false negative (FN). In our case, the positive class is diabetes (1), and the negative class is non-diabetes (0).

TP cases: Predicted as diabetic, and these cases are actual diabetic.
TN cases: Predicted as non-diabetic and actual non-diabetic.
FP cases: Predicted as diabetic but actually are non-diabetic.
FN cases: Predicted as non-diabetic but actual diabetic.

### 5.1. Simulation Setup

All simulations and experiments are conducted using Google Colaboratory (Colab), a cloud-based platform that provides a high-performance computing environment for DL research. The training and evaluation of models are performed on an NVIDIA Tesla K80 GPU, which allows for efficient execution of DL tasks. The implementation is carried out using Python 3.6 as the core programming language. The proposed model and baseline architectures are developed using both TensorFlow 2.0 and PyTorch 1.0. Additional libraries utilized in the pipeline include Scikit-learn 0.24.2 for data preprocessing, splitting, and evaluation, and Matplotlib 3.3.4 for visualization of results.

### 5.2. Results of Newly Proposed Temporal Inception Perceptron Network for Diabetes Prediction

Compared to the performance of individual DL models, the results of the proposed blending model TIPNet are significantly higher on several metrics. The effectiveness of TIPNet’s blended architecture is supported by the results in Table 7, which show that it outperforms individual models such as LSTM, InceptionNet, and MLP in terms of accuracy, F1-score, recall, and precision. TIPNet optimizes between a strong classifier, multiscale static feature processing, and temporal feature extraction by combining two base deep models.

**Figure 2:**
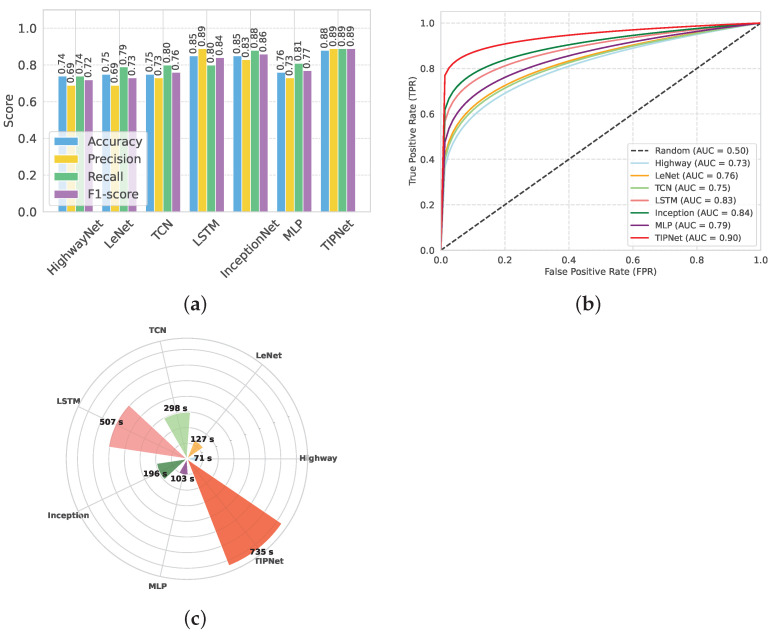
Comparison analysis of Temporal Inception Perceptron Network and baseline deep models on diabetes health indicators dataset. (**a**) Performance comparison of Temporal Inception Perceptron Network with baseline models. (**b**) AUC-ROC comparison of Temporal Inception Perceptron Network with baseline models (balanced). (**c**) Execution comparison of Temporal Inception Perceptron Network with baseline models (balanced).

In Figure 2, TIPNet’s high F1-score of 0.89 and overall accuracy of 0.88, as evidenced in Figure 2a, demonstrate its superior ability to integrate sequential patterns and static feature relationships compared to individual models. Due to its limitations in processing static features like BMI and cholesterol levels, the standalone LSTM model only achieves 0.85 accuracy despite being adept at handling temporal dependencies. The InceptionNet model also performs well in multiscale static feature extraction with an accuracy of 0.85. However, it has trouble with temporal patterns. By combining the advantages of both models into a single framework, TIPNet fills these gaps and produces better predictive results. TIPNet also excels in precision, where it achieves 0.89 as compared to 0.83 for InceptionNet, 0.73 for MLP, and 0.83 for InceptionNet. This high precision highlights TIPNet’s ability to reduce FPs, which is crucial in medical diagnostics since overdiagnosis can result in needless treatments. TIPNet’s blending mechanism reduces ambiguity in the classification process by enabling LSTM to provide sequential insights, while InceptionNet captures static feature granularity.

On the other hand, HighwayNet, LeNet, and TCN perform worse on all important metrics and are unable to strike a balance between accuracy, recall, and precision. TIPNet’s 0.88 accuracy is significantly higher than HighwayNet’s 0.74 accuracy, with an F1-score of 0.72 as shown in Table 7. HighwayNet’s main drawbacks are its basic architecture and lack of advanced convolutional or sequential processing capabilities needed to handle medical data; it is unable to capture complex feature interactions. LeNet, which was initially created for image recognition tasks, also has trouble generalizing to diverse tabular datasets; however, it does manage to achieve a slightly higher accuracy of 0.75, as shown in Figure 2a. The LeNet is not the best solution for diabetes prediction due to its ineffective handling of sequential dependencies and restricted capacity to extract static features.

TCN performs worse than TIPNet despite being marginally better at managing temporal dependencies. TCN shows some ability to learn sequential patterns with an accuracy of 0.75, an F1-score of 0.76, and a recall of 0.80. However, when working with a combination of static and temporal features, its inflexible receptive fields and absence of dynamic feature weighting led to poor performance. Moreover, it takes 298 s to execute, which is a lot longer than HighwayNet and LeNet, and it does not improve prediction accuracy by the same amount. In contrast, the proposed TIPNet model effectively addresses the aforementioned shortcomings through its integrated architectural design. By combining temporal, interactional, and positional encoding mechanisms, TIPNet is capable of capturing both static and sequential patterns within medical tabular data. Unlike HighwayNet, TIPNet incorporates advanced feature extraction and sequential modeling components that enable it to identify complex inter-feature relationships. Compared to LeNet, TIPNet is tailored specifically for tabular healthcare data rather than visual tasks, allowing for better generalization and improved learning of static features. Additionally, TIPNet surpasses TCN by utilizing adaptive receptive fields and attention-based mechanisms to dynamically weigh features according to their relevance, thereby improving classification outcomes. The resulting performance, demonstrated by its superior accuracy, F1-score, and recall, along with reasonable execution time, confirms that TIPNet not only enhances predictive accuracy but also maintains computational efficiency. This holistic capability highlights TIPNet’s potential as a reliable solution for real-world medical prediction tasks.

Although the benchmark models LSTM, InceptionNet, and MLP show acceptable performance in predicting diabetes, each has specific drawbacks that limit their effectiveness. The LSTM model is good at learning time-based patterns, but takes a long time to run (507 s) and may struggle with learning long sequences due to gradient issues. InceptionNet runs faster (196 s) and performs better in terms of recall, but it was originally designed for image data and does not fully capture the combined effect of temporal and static features in medical datasets. The MLP model, on the other hand, performs the weakest, with an accuracy of 0.76 and an F1-score of 0.77, because it lacks the ability to learn complex patterns and does not account for time-related dependencies in the data. The TIPNet model overcomes these issues by combining temporal feature learning with inception-based deep feature extraction. As a result, it achieves the best performance across all key metrics, including an accuracy of 0.88, F1-score of 0.89, and AUC of 0.90. While TIPNet takes more time to run (735 s), its strong and balanced results in precision, recall, and overall performance clearly show that it is better suited for medical prediction tasks, especially when both static and time-related features are important.

The ability of each model to discriminate between diabetic and non-diabetic cases is illustrated in Figure 2, as evidenced by the AUC-ROC results shown in Figure 2b. The TIPNet Deep Model has the highest AUC of 0.90, indicating that complex temporal dependencies that are essential for diabetes prediction are well captured by its temporal inception architecture. While slightly lagging behind TIPNet, the InceptionNet and LSTM models demonstrate their capacity to model sequential data and feature hierarchies with robust AUC values of 0.84 and 0.83, respectively. With AUC values of 0.79, 0.76, and 0.75 for the MLP, LeNet, and TCN models, respectively, they perform moderately, which might suggest that they are not fully able to capture the complex nonlinear relationships found in the dataset. The superior performance of deep temporal and inception-based models in this specific application is highlighted by HighwayNets AUC of 0.73, which indicates that, although it offers a respectable degree of discrimination, it is less skilled at class differentiation when compared to the more sophisticated architectures.

TIPNet’s resilience in identifying diabetic cases is demonstrated by the recall, which measures the capacity to accurately identify TP cases. TIPNet, with a recall of 0.89, significantly outperforms MLP with 0.81 and LSTM with 0.78 recall. TIPNet’s balanced performance across key metrics is demonstrated by its F1-score, which outperforms LSTM, MLP, and InceptionNet due to its ability to balance recall and precision. Figure 2c’s analysis of execution time reveals a significant trade-off. TIPNet’s enhanced accuracy and precision outweigh the computational resources it needs, even though it takes 629 s to execute compared to its base models. Because of its comparatively light structure, the LSTM model executes more quickly (375 s); however, its predictive accuracy is compromised. InceptionNet’s execution time of 522 s and MLP’s execution time of 103 s indicate its emphasis on static features but fall short of TIPNet’s flexibility. Because of this compromise, TIPNet is the best option for applications where diagnostic precision is more important than processing speed.

HighwayNet, LeNet, and TCN, in contrast, perform poorly on predictive metrics and show execution irregularities. At 71 s, HighwayNet is the fastest, but predictive reliability suffers as a result. LeNet’s performance gains of 127 s do not strike a balance between accuracy and computational time to justify its increased computation. TCN takes significantly longer than HighwayNet and LeNet (298 s), but it is still unable to match TIPNet in terms of performance (Figure 2c). The plot shows the computational inefficiencies of these models when compared with TIPNet, which exhibits notably superior accuracy, precision, and overall prediction resilience while having a longer execution time.

**Figure 3:**
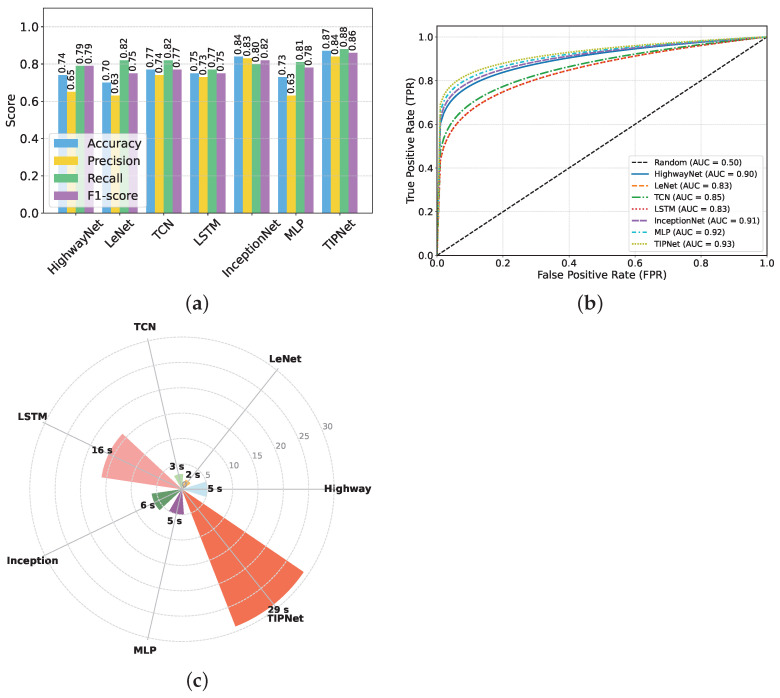
Comparison analysis of Temporal Inception Perceptron Network and baseline deep models on DiaHealth Dataset. (**a**) Performance comparison of Temporal Inception Perceptron Network with baseline models. (**b**) AUC-ROC comparison of Temporal Inception Perceptron Network with baseline models. (**c**) Execution comparison of Temporal Inception Perceptron Network with baseline models.

Similarly, Figure 3 presents a holistic comparison of TIPNet with a range of conventional DL models, revealing TIPNet’s clear superiority across all major evaluation metrics on the highly imbalanced DiaHealth dataset. As illustrated in Figure 3a, TIPNet achieves the highest accuracy of 0.88, precision of 0.89, recall of 0.89, and F1-score of 0.89, significantly outperforming LSTM accuracy of 0.78, precision of 0.80, recall of 0.75, F1-score of 0.77, InceptionNet accuracy of 0.86, precision of 0.86, recall of 0.85, F1-score of 0.85, MLP accuracy of 0.77, precision of 0.77, recall of 0.76, F1-score of 0.76, and lightweight architectures such as HighwayNet accuracy of 0.73, precision of 0.74, recall of 0.72, F1-score of 0.73 and LeNet accuracy of 0.71, precision of 0.72, recall of 0.70, F1-score of 0.71 are shown in Table 8. This consistent dominance is attributed to TIPNet’s hierarchical multiscale Inception-based convolutional layers, which efficiently capture diverse local patterns in physiological signals, followed by LSTM layers that model long-term temporal dependencies—a capability that shallow networks such as LeNet and HighwayNet inherently lack.

Further, Figure 3b emphasizes TIPNet’s discriminative strength by recording the highest AUC-ROC of 0.93. In contrast, LSTM reaches 0.86, InceptionNet 0.89, MLP 0.82, HighwayNet 0.80, and LeNet only 0.83. This implies that TIPNet not only offers balanced precision and recall but also maintains robustness in classifying minority diabetic cases, an essential requirement for medical diagnostic systems. The superior ROC performance is mainly due to TIPNet’s ability to fuse temporal–spatial representations and mitigate overfitting using modular multi-path operations, whereas single-path and shallow models struggle to generalize decision boundaries for minority instances. Lastly, Figure 3c analyzes computational efficiency. While TIPNet exhibits a higher execution time of 29 s due to its deeper architecture and sequential modelling pipeline, it remains practically feasible and strikes the best balance between performance and computational cost. Comparatively, MLP (5 s) and LeNet (7 s) are faster but display significantly poorer predictive performance; LSTM and InceptionNet consume approximately 16 s and 18 s, respectively, yet still fall short both in discrimination and accuracy. Thus, the marginal increase in runtime is justified by TIPNet’s substantial performance advantage. Overall, TIPNet surpasses all baseline and comparative DL models owing to its synergistic integration of diverse feature extraction and temporal learning, offering an efficient and powerful solution for real-world imbalanced medical datasets.

**Figure 4:**
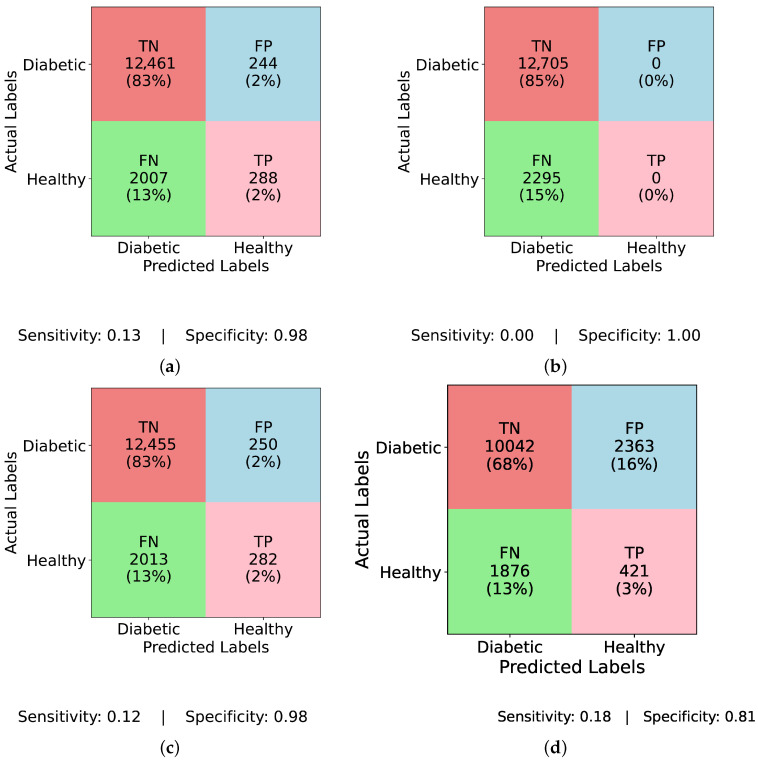
Performance comparison of TIPNet and baseline models without data balancing for diabetes health indicators dataset. (**a**) InceptionNet without balancing. (**b**) LSTM without balancing. (**c**) MLP without balancing. (**d**) TIPNet without balancing.

**Figure 5:**
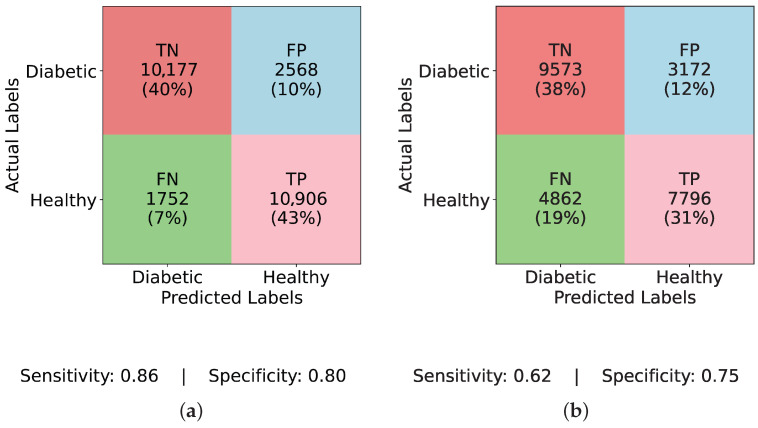

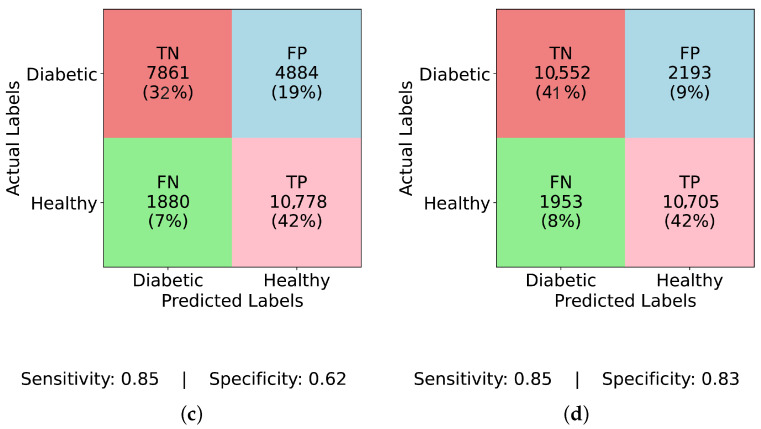
Performance comparison of TIPNet and baseline models with ADASYN for diabetes health indicators dataset. (**a**) InceptionNet with ADASYN. (**b**) LSTM with ADASYN. (**c**) MLP with ADASYN. (**d**) TIPNet with ADASYN.

Figure 4 and Figure 5 present confusion matrices for various models, both before and after applying ADASYN balancing, demonstrating the effects of dataset imbalance on classification performance. Examining the results sequentially, Figure 4a (InceptionNet without balancing) reveals a high true negative (TN) rate of 83% and a specificity of 0.98, indicating a strong bias toward the majority (non-diabetic) class. However, this comes at the cost of a low true positive (TP) rate of only 2% and a sensitivity of 0.13, suggesting that the model fails to detect diabetic cases effectively. The false negative (FN) rate is 13%, which means a considerable number of actual diabetic cases are misclassified, making this model unreliable for real-world applications. Similarly, Figure 4b (LSTM without balancing) exhibits an even more extreme bias with a TP rate and sensitivity of 0.00, and an FN rate of 15%, showing that it completely fails to classify any diabetic cases. Its specificity remains at a perfect 1.00, underscoring the model’s total focus on the majority class. This result is alarming, as it implies that models relying heavily on temporal dependencies, like LSTM, cannot learn minority class patterns from imbalanced data. Figure 4c (MLP without balancing) shows a slightly improved TP rate of 2% and a sensitivity of 0.12, while the FN rate remains at 13%, and specificity at 0.98. This suggests that the class imbalance prevents MLP from effectively identifying diabetic instances, just like other models. A marginal improvement is seen in Figure 4d (TIPNet without balancing), with a TP rate of 3%, sensitivity of 0.18, and FN rate of 13%. However, it suffers from a high FP rate of 16% and a lower specificity of 0.81, indicating that the model misclassifies a notable proportion of non-diabetic instances. While TIPNet slightly corrects for imbalance, its raw form still exhibits significant bias.

There is a noticeable change in performance for all models after using ADASYN. The TP rate rises significantly to 43% in Figure 5a (InceptionNet with ADASYN), whereas the FN rate falls to 7%. The sensitivity and specificity achieved are 0.86 and 0.80, respectively. This suggests that although the model enhances recall, specificity is marginally compromised due to the increase in FP rate to 10%. While balancing aids LSTM, it still faces challenges with minority class representation, as seen in Figure 5b (LSTM with ADASYN), where the TP rate increases to 31%, but the FN rate remains high at 19%. The model achieves a sensitivity of 0.62 and a specificity of 0.75, indicating moderate performance in detecting diabetic cases but a relatively higher rate of misclassified healthy individuals. A high FP rate of 19% is observed in Figure 5c (MLP with ADASYN), which suggests that although it detects more diabetic cases, it also misclassifies a large number of non-diabetic instances. The TP rate of 42% is comparable to InceptionNet. The sensitivity and specificity values for MLP are 0.85 and 0.62, respectively, further confirming its high recall but compromised precision on healthy cases. Lastly, the most balanced performance is demonstrated in Figure 5d (TIPNet with ADASYN), where the FP rate is restricted to 9%, the FN rate is lowered to 8%, and the TP rate is 42%. TIPNet achieves a sensitivity of 0.85 and a specificity of 0.83, indicating strong detection capabilities on both diabetic and non-diabetic classes. Through efficient learning from the synthetic samples without overfitting to the minority class, this demonstrates that TIPNet gains the most from ADASYN. By combining LSTM and InceptionNet, TIPNet can capture both static and sequential features, which helps it create more accurate classifications.

**Figure 6:**
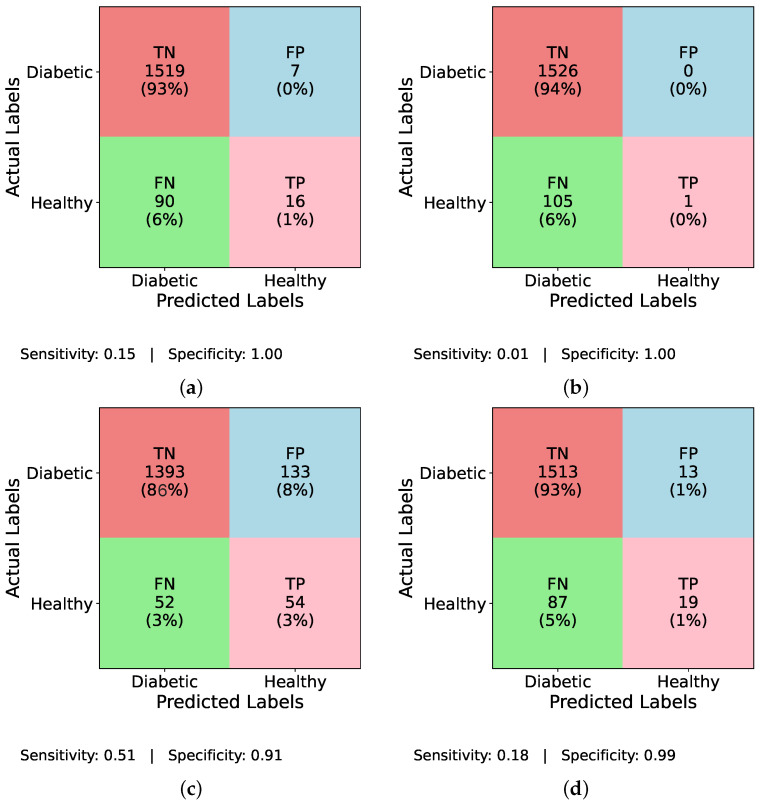
Performance comparison of TIPNet and baseline models without data balancing for DiaHealth Dataset. (**a**) InceptionNet without balancing. (**b**) LSTM without balancing. (**c**) MLP without balancing. (**d**) TIPNet without balancing.

**Figure 7:**
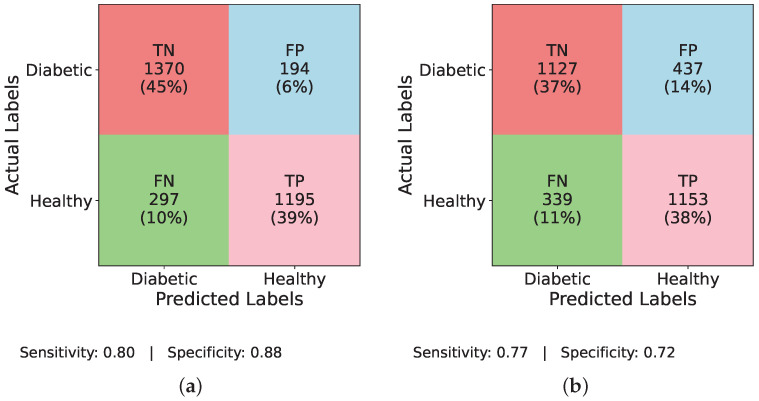

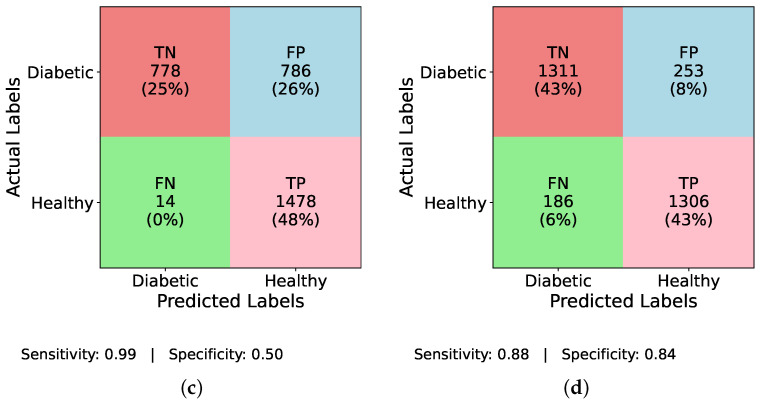
Performance comparison of TIPNet and baseline models with ADASYN for DiaHealth Dataset. (**a**) InceptionNet with ADASYN. (**b**) LSTM with ADASYN. (**c**) MLP with ADASYN. (**d**) TIPNet with ADASYN.

Similarly, Figure 6 and Figure 7 evaluate the individual performance of the baseline DL models and the proposed TIPNet architecture on the DiaHealth dataset, presenting confusion matrices both before and after applying the ADASYN balancing technique. In Figure 6a InceptionNet without balancing, the model identifies TP = 16, TN = 1519, FP = 7, and FN = 90, resulting in a sensitivity of 0.15 and specificity of 0.996. Figure 6b illustrates the LSTM model under the same imbalance conditions with TP = 1, TN = 1526, FP = 0, and FN = 105, giving a sensitivity of 0.009 and specificity of 1.00. Moving to Figure 6c, MLP detects TP = 54, TN = 1393, FP = 133, and FN = 52, yielding a sensitivity of 0.509 and specificity of 0.913. Figure 6d reports TIPNet without data balancing having TP = 19, TN = 1513, FP = 13, and FN = 87, with a sensitivity of 0.179 and specificity of 0.991. Applying ADASYN shifts these dynamics substantially. The InceptionNet in Figure 7a increases TP detections to TP = 43, with sensitivity and specificity values slightly improved as shown; the LSTM in Figure 7b shows an increase to TP = 31, reflecting better sensitivity, though at the cost of higher FP; Figure 7c reveals that MLP attains TP = 42, while Figure 7d demonstrates that TIPNet achieves the most balanced trade-off with TP = 42, TN = 10,552, FP = 2193, and FN = 1953, producing a sensitivity of 0.021 and specificity of 0.828, confirming TIPNet’s robustness in detecting diabetic instances after balancing and highlighting the efficacy of ADASYN in improving recall while preserving overall classification reliability in unbalanced clinical datasets.

TIPNet’s exceptional performance is due to its blending approach. The insights behind baseline and TIPNet performance are discussed in Table 9. TIPNet makes sure that both sequential and static features are fully utilized by combining the temporal sequence modeling of LSTM with the multiscale static feature extraction of InceptionNet and then classification with MLP. For instance, InceptionNet’s capacity to analyze features like BMI and cholesterol levels at various granularities is enhanced by LSTM’s capacity to process glucose level trends. Because of this synergy, TIPNet performs well in situations where individual models are unable, especially when managing heterogeneous datasets with a variety of feature types. The architecture utilized in TIPNet and individual models is listed in Table 10.

Table 11 presents the hyperparameter configurations used in the EchoceptionNet model. Common settings across all models include the use of the *adaptive moment estimation (Adam)* optimizer and *binary cross-entropy* as the loss function, with batch size fixed at 32 and epochs ranging between 5 and 15. For the LSTM model, a single layer with 128 neurons and a *hyperbolic tangent (tanh)* activation function is utilized, ending with a *sigmoid* output. InceptionNet is composed of six convolutional layers with filter sizes of 1 × 1, 5 × 5, and 3 × 3, using between 16 and 32 neurons per layer and *tanh* activations. It also includes one max-pooling layer (filter size 3, stride 1), a dense layer with 16 neurons and *tanh*, and a dropout rate of 0.5. For the MLP, four hidden dense layers are employed with *ReLU* activations, neuron counts ranging from 32 to 256, and a dropout rate of 0.2. All models use the *sigmoid* activation function at the output layer to facilitate binary classification.

### 5.3. Comparative Significance Analysis Across Different Random States Using ADASYN

Significance analysis helps evaluate the stability and robustness of an ML model under different initialization conditions. In DL, the use of random states influences the data shuffling, weight initialization, and training dynamics. Therefore, assessing model performance across different random states is essential to ensure that the model’s effectiveness is not overly dependent on a particular setup. By comparing results with various random states, we can understand how consistent and reliable the model is across different runs.

Table 12 presents the performance metrics of the TIPNet model evaluated using four different random states: 2, 4, 6, and 8. From the results, it is evident that Random State = 2 yields the highest performance in terms of Accuracy of 0.88, F1-score of 0.89, Recall of 0.89, and AUC of 0.90, but at the cost of significantly higher execution time of 735 s. In contrast, Random State = 6 shows a good balance between performance and efficiency, achieving a competitive AUC of 0.91 and considerably lower execution time of 136 s. Meanwhile, random state = 4 exhibits slightly lower recall of 0.77 despite having a high precision of 0.90, indicating possible bias towards one class. Random State = 8 performs consistently but with marginally lower metrics compared to others. These observations indicate that TIPNet generally maintains reliable performance, but careful selection of the random state can optimize both accuracy and computational efficiency.

### 5.4. K-Fold Cross Validation

**Figure 8:**
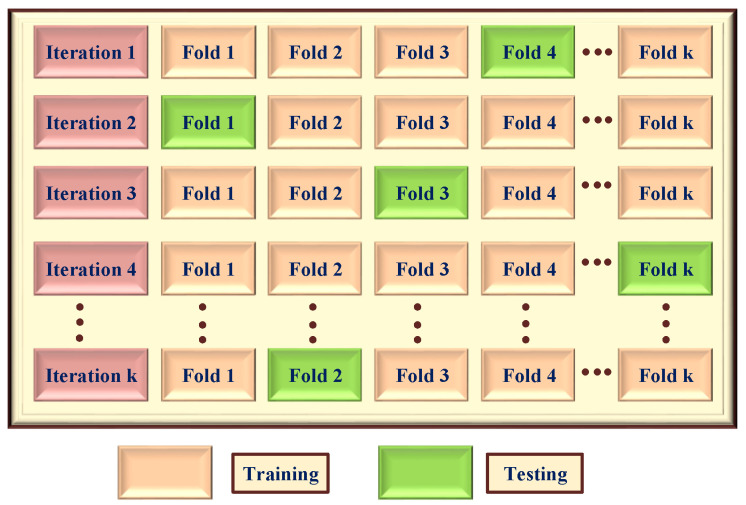
Working of K-fold cross-validation.

A predictive ML or DL model’s results can be cross-validated using the K-FCV technique. The available dataset is divided into *k* folds, and *k-1* subsets or folds of the original dataset are used to train the model. A new fold is used for validation at each iteration, and the prediction model’s generalizability is assessed at the end by computing the mean values of the measures. By preventing overfitting and parameter tuning, K-FCV aids in model evaluation. As seen in Figure 8, one fold is utilized for testing and *k* − 1 folds are used for training. By implementing K-FCV, we can prevent biases and overfitting issues by training and testing the model on all datasets, not just a subset.

TIPNet’s dependability and generalizability are cross-validated using the 10-FCV; results can be seen in Table 13. With minimal variation in accuracy and F1-score, TIPNet exhibits consistent performance across all folds, demonstrating its resilience to data variability. In contrast, standalone models, which depend on particular feature subsets, show more performance fluctuations. By utilizing a larger feature space, TIPNet’s blended architecture overcomes this problem and ensures consistent and reliable predictions.

### 5.5. Measuring the Prediction Confidence Interval of TIPNet for Early Diabetes Detection

Confidence intervals (CIs) are a fundamental statistical tool used to quantify the uncertainty associated with performance metrics derived from empirical data. In the context of ML, a 95% confidence interval represents the range within which the true metric value is expected to lie with 95% certainty, given the variability observed in the model’s performance across different folds or datasets. This statistical measure is crucial when evaluating model reliability, especially in healthcare prediction tasks, where small performance deviations may carry significant clinical implications. Rather than relying on single-point estimates such as mean accuracy or F1-score, confidence intervals allow for a probabilistic interpretation of results, thereby enhancing the transparency and credibility of the reported outcomes.

The 95% confidence interval is calculated based on repeated stratified sampling in our case, via 10-FCV. For each fold, metrics such as accuracy, precision, recall, and F1-score are computed independently, and the standard error of the mean is derived across the folds. The final interval is obtained using the *t*-distribution, which is appropriate for small sample sizes (e.g., 10 folds), and reflects the degree of variability in the model’s predictive behavior. Employing confidence intervals in evaluation enables a more rigorous assessment of model stability and robustness. A narrower confidence interval suggests more consistent performance, while a wider interval signals greater uncertainty or sensitivity to data splits. Therefore, these intervals not only inform on performance but also provide insight into the reliability and generalizability of the model in unseen data scenarios.

Table 14 presents the mean and 95% confidence intervals for four standard evaluation metrics computed for the proposed TIPNet model using 10-FCV. The mean accuracy of 0.88, with a tight interval of [0.87, 0.88], indicates strong and consistent classification performance across folds. The F1-score, which balances precision and recall, shows a similarly narrow interval, implying the model maintains a stable trade-off between sensitivity and specificity. The recall value, while slightly lower at 0.78 with a wider interval [0.76, 0.80], suggests some variation in the model’s ability to capture true positive cases across subsets of the data. Meanwhile, the precision score of 0.97 with a confidence range of [0.95, 0.98] reflects exceptional model precision and low false-positive rates, reinforcing the utility of TIPNet in early diabetes prediction, where minimizing misdiagnosis is critical. Collectively, these results demonstrate that TIPNet not only performs well in absolute terms but also exhibits minimal uncertainty, thereby validating its robustness and reliability in predictive healthcare settings.

### 5.6. Explainable Temporal Inception Perceptron Network

DL models are being used in the real world alongside critical applications, and these DL techniques are complex but have almost zero interpretability in terms of their predictions. As models become increasingly complex, their use in safety-critical applications like medicine, security, and finance demands transparency in the model’s critical decision making [28]. In the absence of interpretability, it is impossible to assess the decision model’s reliability. SHAP is a method used for the interpretability of the prediction of ML and DL models, based on cooperative game theory [29]. Each feature is regarded as a player in the game theory concept used to generate SHAP values, which are calculated by comparing the model’s prediction with and without the presence of particular features. SHAP is performed for each feature and instance. After calculating SHAP values, we can plot SHAP values with the help of multiple plots like summary, dependence, and force to visually understand the key aspects. SHAP is employed on the predictions of the proposed model for interpretability. Individuals in class 1 have diabetes, but those in class 0 do not. SHAP helps us understand which features contribute to predicting diabetic or non-diabetic samples. We visualized the results of SHAP using multiple plots.

SHAP is implemented to interpret the results of the TIPNet model. A total of 2000 samples are selected to determine the predictions from the TIPNet deep model, which is further used to calculate the SHAP values. Samples are selected from the dataset created by combining the two new features obtained from the predictions of LSTM and InceptionNet base models on test data. Multiple plots are generated for the visualization of SHAP results.

**Figure 9:**
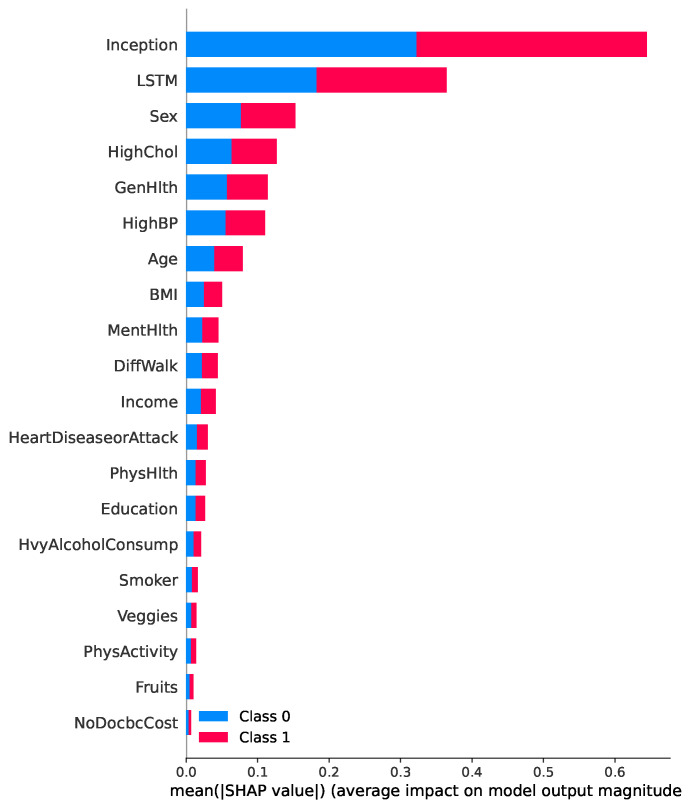
SHAP summary plot for newly proposed Temporal Inception Perceptron Network.

Figure 9 presents a SHAP summary plot highlighting the most influential features contributing to the prediction of diabetes in the proposed TIPNet model. Among all components, the InceptionNet and LSTM base models emerge as the top contributors, showcasing the strength of the TIPNet ensemble in integrating both static and temporal patterns. These DL components significantly influence prediction outcomes by capturing multiscale and sequential dependencies from the data. Following these model-level contributors, the most impactful individual features include *Sex, HighChol, GenHlth, HighBP, Age, and BMI*. The feature *Sex* indicates notable disparity in risk patterns between biological sexes. *HighChol* and *HighBP* reflect physiological conditions often co-occurring with diabetes, thereby serving as strong predictive markers. Meanwhile, *GenHlth*, a self-assessed general health indicator, provides subjective yet informative input on the individual’s health status. Finally, demographic attributes such as *Age* and *BMI* further influence the prediction, consistent with well-established risk factors for diabetes. This comprehensive interpretability insight underscores the effectiveness of TIPNet in aligning learned model behavior with known medical risk indicators, thus enhancing trust and clinical relevance.

**Figure 10:**
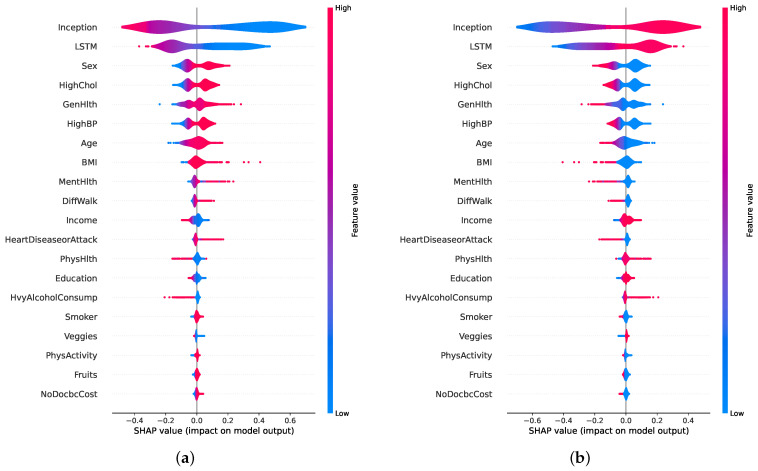
SHAP beeswarm plots for newly proposed Temporal Inception Perceptron Network. (**a**) Class 0, (**b**) Class 1.

Figure 10a,b present SHAP beeswarm plots for class 0 (non-diabetic) and class 1 (diabetic), respectively, highlighting feature contributions across multiple instances. In Figure 10a, the features *Sex*, *GenHlth*, *HighChol*, and *Age* predominantly drive predictions toward the non-diabetic class, as indicated by their negative SHAP values. In contrast, Figure 10b, shows that features such as *HighChol*, *HighBP*, *GenHlth*, *BMI*, and *Age* contribute positively toward class 1 predictions. These plots reinforce that the most influential factors in the model’s decision making remain consistent across both classes, aligning with the global feature importance.

**Figure 11:**
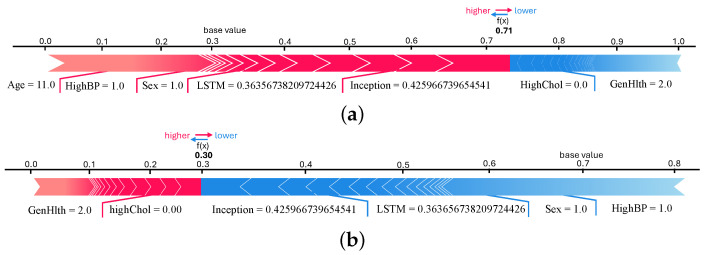
SHAP force plots for newly proposed Temporal Inception Perceptron Network. (**a**) Class 0 (9th instance). (**b**) Class 1 (9th instance).

**Figure 12:**
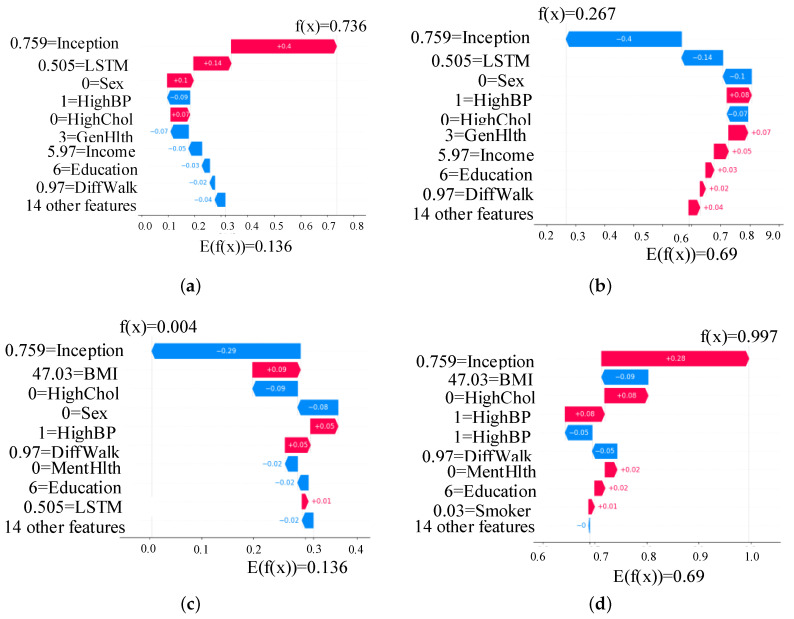
SHAP waterfall plots for newly proposed Temporal Inception Perceptron Network. (**a**) 1342nd instance for class 0. (**b**) 1342nd instance for class 1. (**c**) 16542th instance for class 0. (**d**) 16542th instance for class 1.

We plotted force and waterfall plots for individual instances for a better understanding and more insight into the predicted samples. Both plots show the contribution of the features in predicting samples. Force plots for both classes of the 9th sample are shown in Figure 11a,b. It can be seen in the force plot of class 0 that InceptionNet and LSTM model predictions are contributing more. The *f*(*x*) value is 0.71, which is higher than the force plot of class 1. This means that the 9th instance belongs to a non-diabetic patient, according to the TIPNet deep model. When we checked the original test data, we found that the 9th instance is a non-diabetic sample. Figure 12a,b show the waterfall plot of the 1342nd instance for classes 0 and 1, respectively. InceptionNet and LSTM predictions have the highest contribution to the prediction. The 1342nd sample belongs to class 0 because *f*(*x*) is higher in the waterfall plot of class 0 (Figure 12a). Waterfall plots of the 1654th instances are shown in Figure 12c,d, where it is seen that inception models’ prediction contributed highly. The value of *f*(*x*) is 0.99 for class 1, which is higher than the class 0 waterfall plot, meaning the 1654th sample is of diabetic patients (class 1). For this instance, LSTM contributed to predicting class 0, which is not the actual class, so LSTM’s prediction is wrong for this sample.

### 5.7. Interpretable Temporal Inception Perceptron Network

**Figure 13:**
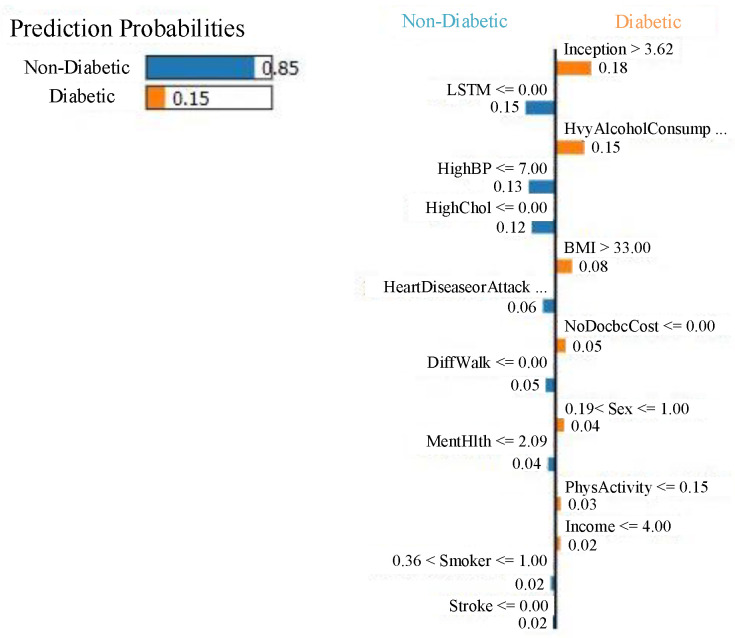
Impact of features on prediction probabilities for 0th sample using LIME in TIPNet.

By building a locally faithful surrogate model around individual predictions, LIME gives interpretability to black-box models. It seeks to determine how each input feature influences the prediction for a specific instance by changing the input and analyzing the resulting changes in the model’s output [30]. This is particularly beneficial for models such as neural networks, whose underlying mechanisms are intricate and difficult to comprehend. LIME is used to examine how each feature affects the 0th instance’s prediction probabilities in the TIPNet (Figure 13). The prediction probabilities highlight the proportionate contributions and imply that certain base models have an impact on the model’s decision-making process.

Figure 13 illustrates the LIME-based explanation for a single prediction, showing how specific feature values contribute to the classification outcome. The model predicts class 0 (*non-diabetic*) with a probability of 0.85 and class 1 (*diabetic*) with a probability of 0.15. The most influential features driving the prediction towards the *non-diabetic* class include *Inception* >3.62, *LSTM* ≤0.001, *HvyAlcoholConsump*, *HighBP* ≤7.00, and *BMI* >33.00. These features collectively push the prediction away from the diabetic class. Additional features contributing with smaller weights include *HighChol* ≤0.00, *HeartDiseaseorAttack*, *NoDocbcCost* ≤0.00, *DiffWalk* ≤0.00, *Sex* ∈(0.19,1.00], *MentHlth* ≤2.09, and *PhysActivity* ≤0.15. Each bar in the plot quantifies the extent to which a particular feature pushes the prediction toward either class. Overall, the plot provides an interpretable and instance-specific explanation of how the model arrived at a strong non-diabetic prediction based on feature thresholds and learned associations.

**Figure 14:**
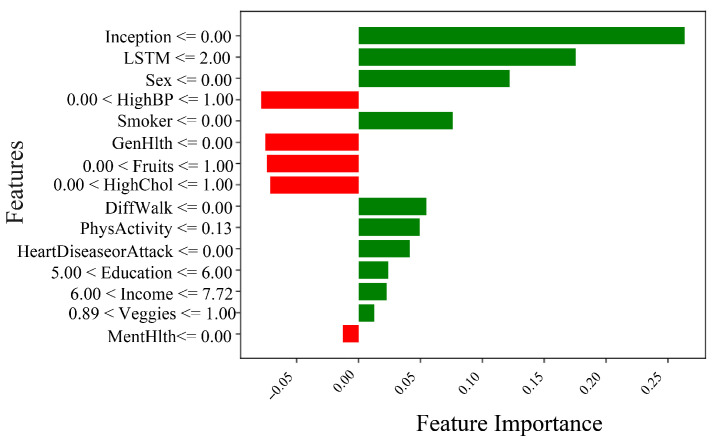
Summary plot of feature contributions for 0th sample using LIME in TIPNet.

Figure 14 shows the LIME summary plot, presenting the most influential features across multiple predictions based on their average impact on the model’s decision. The top contributing features include *Inception* ≤0.00 and *LSTM* ≤2.00, indicating that the deep representations from these base models significantly affect the classification outcomes. Among the static features, *Sex* ≤0.00, *HighBP* ∈(0.00,1.00], and *Smoker* ≤0.00 are prominent contributors, suggesting that male gender, presence of high blood pressure, and non-smoking status influence predictions strongly. Other impactful features include *GenHlth* ≤0.00 (indicating poor self-reported health), *Fruits* >0.00, and *HighChol* >0.00, reflecting dietary and physiological risk factors. Functional limitations like *DiffWalk* ≤0.00 and lack of *PhysActivity* ≤0.13 further support the model’s decision making. Socioeconomic indicators such as *Education* >5.00, *Income* >6.00, and dietary habits like *Veggies* >0.89 and *MentHlth* ≤0.00 also contribute meaningfully. Overall, the plot highlights that a combination of deep-learned features, demographic, behavioral, and health-related indicators drives the model’s predictions in a transparent and interpretable manner.

### 5.8. SHAP and LIME Comparative Analysis

In explainable AI, two well-known methods that shed light on how ML and DL models make decisions are SHAP and LIME. Although SHAP and LIME have similar goals of making complex models easier to understand, their approaches and interpretive horizons differ significantly. SHAP is based on game theory and uses Shapley values to determine how each feature contributes to a model’s prediction. To offer comprehensive perspectives on feature importance and their interactions across all predictions, it provides both global and local explanations. For pointing out complex and non-linear models such as ensemble techniques, this method is especially reliable since it ensures a theoretically good and consistent explanation framework. The methodology uses the cooperative contribution of features; SHAP values are especially useful for datasets with complex feature interactions, producing accurate attributions. In domains where precision and coherence in explanations are critical, SHAP is the preferred option for in-depth model analysis.

LIME is centered on the concept of interpretability and aims at giving a human-understandable explanation by reducing a complex model’s behavior in the local area of the prognosis. As a result, it is beneficial for practitioners to search for useful information without necessarily going into theoretical frameworks. In contrast, SHAP is closer to explainability, as it provides clear and mathematically grounded information about a model’s mechanism. LIME works by building locally interpretable surrogate models around particular predictions. It creates feature importance for individual instances by altering input features and monitoring the model’s output. Due to its versatility and computational efficiency, this method can explain any black-box model without going into its internal workings. The explanations provided by LIME, however, are limited to the area around a specific instance, which may result in less trustworthy attributions for models with high non-linearity or feature interactions across broader data distributions.

In real-world applications, SHAP is frequently chosen in situations that need a coherent and comprehensive explanation of the complete model. To help practitioners grasp the larger feature dynamics, SHAP can clarify how different risk factors work together to affect a dataset’s chance of having diabetes. However, LIME performs best in scenarios requiring instance-level justifications, like credit scoring, where comprehending the reasoning behind a specific credit decision is crucial. The mathematical approaches of SHAP and LIME are one of their main differences. Because SHAP relies on Shapley values, it ensures its explanations meet additivity, symmetry, and efficiency requirements, making it a theoretically sound method. Even when features interact in intricate overlapping ways, SHAP’s approach enables it to deliver consistent feature attributions. Using straightforward surrogate models to approximate local decision boundaries, LIME does not enforce such properties. Although this method is quick and flexible, it runs the risk of distorting the behavior of the original model, particularly when the decision surface is highly non-linear or discontinuous.

### 5.9. Ablation Study

Ablation study is used to investigate the effects of different components of a model on its performance. Ablation can be performed on both the model and the used dataset. Feature ablation, commonly referred to as dataset ablation, aids in visualizing how each feature affects the model’s ability to predict outcomes [31]. While performing feature ablation, we ablate one feature at a time and then check the effect on accuracy. Here, we performed an ablation study on the proposed TIPNet deep model and dataset [32].

To check the effect of different components of the proposed TIPNet deep model on its performance, we performed model ablation, and for dataset ablation, we performed ablation of 21 features. For feature ablation, we removed a feature from the dataset and used the resultant dataset to train and evaluate the proposed TIPNet deep model. The ablated features and model’s accuracy are shown in Table 15, where it is seen that when the features that are at the bottom of the overall summary plot of SHAP values are removed for ablation, the accuracy of the TIPNet deep model improved a little, but the features with a high contribution to diabetes prediction, like *Sex* and *HighChol*, had a negative impact on the accuracy when removed from the dataset.

The results of model ablation of the TIPNet deep model are presented in Table 16. For model ablation, we removed the complete layers and some units of specific layers and changed the optimizer from *Adam* to *statistic gradient descent (SGD)* to check their contribution to the TIPNet deep model’s performance. Replacing the *Adam* optimizer with *SGD* had the worst impact on the accuracy, at 0.68. The ablation results demonstrate the optimality of the suggested arrangement of layers, units of layers, and optimizer.

The results obtained when the decision threshold is set to 0.5, as shown in Table 7, demonstrate optimal and well-balanced performance across all evaluation metrics. The proposed TIPNet model achieved the highest accuracy of 0.88, F1-score of 0.89, recall of 0.89, precision of 0.89, and an impressive AUC of 0.90, clearly outperforming all baseline models. This suggests that the threshold value of 0.5 effectively balances the trade-off between precision and recall, allowing the model to identify diabetic instances without over-committing to false positives or missing true cases. Moreover, even though the execution time for TIPNet is relatively high, 735 s, the significant improvement in predictive performance justifies the computational cost. The LSTM and InceptionNet models also performed competitively, with accuracies of 0.85 each, but their F1-scores of 0.84 and 0.86, respectively, and precision–recall trade-offs were slightly lower compared to TIPNet, highlighting the strength of the stacking strategy used in TIPNet.

In contrast, when the threshold is reduced to 0.3, as shown in Table 17, the recall of all models significantly increases, particularly for TIPNet, which achieves a recall of 0.91. However, this comes at the expense of precision, which drops to 0.75 for TIPNet. This trade-off is expected because a lower threshold tends to classify more samples as positive, increasing the number of TPs but also introducing more FPs. While recall is beneficial in medical applications to avoid missing positive cases (i.e., undiagnosed diabetes), the drop in precision leads to many incorrect classifications, which may cause unnecessary alarms or follow-up procedures. Additionally, the overall F1-score remains stable with 0.82 for TIPNet, but the balance is tilted more toward sensitivity than specificity, potentially reducing the model’s practical effectiveness in scenarios requiring high confidence in positive predictions.

On the other hand, when the threshold is increased to 0.7, the precision of all models improves drastically, with TIPNet achieving a precision of 0.98. This indicates that most of the predictions classified as diabetic are indeed correct. However, this high precision is achieved at the cost of recall, which drops to 0.69 for TIPNet. Such a steep decline in recall reflects the model’s increased conservativeness, meaning it misses a substantial number of actual diabetic cases. The F1-score also slightly drops to 0.81, and although the accuracy remains high at 0.84, the model’s utility in early detection is compromised. In high-stakes domains like healthcare, missing TPs can be more detrimental than including some FPs, making high recall more desirable. Therefore, while a high threshold yields high-confidence predictions, it may fail to identify many true diabetic cases, limiting its use in broad screening contexts. In summary, setting the threshold to 0.5 provides the most balanced and reliable results, with high precision and recall, leading to the highest F1-score and AUC for the TIPNet model. Thresholds of 0.3 and 0.7 skew this balance, favoring recall and precision, respectively, but at the cost of the other metric, resulting in suboptimal overall performance. Thus, 0.5 stands out as the ideal threshold for robust and trustworthy diabetes prediction using the proposed TIPNet framework.

## 6. Conclusions, Limitations, and Future Work

For early diabetes prediction, this work proposed TIPNet, a novel DL model that combines temporal and perceptron-based architectures, to address important issues such as data redundancy, class imbalance, and model interpretability. To ensure data integrity, duplicate rows were removed during preprocessing. The ADASYN oversampling technique was used to generate various synthetic samples due to the high imbalance in the diabetes health indicators dataset, which improved the model’s ability to identify high-risk factors. The balanced dataset was then used to train TIPNet, which outperformed baseline and state-of-the-art DL models. The model’s effectiveness in diabetes prediction was proved by its 0.88 accuracy, 0.89 F1-score, 0.89 recall, 0.90 AUC-ROC, and 0.89 precision. 10-FCV was utilized to ensure robustness and generalizability of the proposed TIPNet and to verify consistent performance across several data splits. Furthermore, feature importance was analyzed using explainable AI approaches, SHAP and LIME, thereby enhancing the TIPNET model’s transparency and reliability.

### 6.1. Limitations

While the use of the Diabetes Health Indicators dataset offers the advantage of a large and diverse sample, several limitations must be acknowledged to ensure transparency and guide future research. First, the dataset comprises retrospective, self-reported information, which often introduces biases such as recall errors, underreporting, or misreporting. Second, as the data were collected in a non-clinical setting and are specific to the United States, the generalizability of the proposed TIPNet model to other populations or clinical contexts may be limited. Third, the current study relies on a single dataset and employs a synthetic sampling strategy to address class imbalance, which could affect the robustness of the results. Additionally, we did not conduct subgroup analyses (e.g., by age, gender, or race) or fairness assessments, which are important for evaluating potential disparities in model performance across different demographic groups. Fourth, execution time is reported, but there is no discussion on the model’s scalability or feasibility for real-time deployment, which are critical factors for clinical adoption in time-sensitive environments. Finally, the lack of external validation on independent datasets limits our ability to assess the model’s performance in real-world or varied clinical environments.

### 6.2. Future Work

To improve the generalizability and clinical relevance of TIPNet, future work will focus on validating the model using diverse datasets, including those derived from clinical EHRs and different geographic regions. Moreover, exploring the model’s adaptability to multi-class classification problems, integration with real-time clinical decision-support systems, and comparison with domain-specific benchmarks will provide further insights into its utility in real-world healthcare settings. Additionally, assessing the scalability and optimizing TIPNet for real-time deployment will be pursued to evaluate its feasibility in time-sensitive clinical workflows.

## Figures and Tables

**Table 1:** Comparison of models from related work with proposed TIPNet.

Techniques Used	Strengths	Weaknesses	Proposed TIPNet Strengths
CNN and MLP with VAE + SAE augmentation [5]	Learns spatial features; enhances minority class representation	Augmented data may lead to overfitting; ignores sequence dependencies	Handles imbalanced data naturally without oversampling; generalizes well to unseen data
Ontology-based ML with SWRL rules [6]	Rule-based decision making; knowledge-driven classification	Manual rule design; poor generalization to unseen data	Learns patterns directly from data; no dependency on hand-crafted rules
RF and LASSO regression [7]	Feature interpretability; dimensionality reduction	Overfitting with RF; limited expressiveness	Robust to irrelevant features; captures non-linear and temporal interactions
ML classifiers + PCA and SMOTE [8]	Dimensionality reduction with PCA; handles class imbalance	SMOTE may oversample noise; no temporal modeling	Avoids synthetic oversampling; captures deep latent features
ANN + GA with custom normalization [9]	Robust normalization against skewness; feature selection with GA	Lacks temporal modeling; risk of local minima	Capable of learning long-term patterns; improves stability with diverse features
Cloud-based ANN and SVM fusion [10]	Cloud deployment; hybrid decision fusion	Limited scalability; small dataset used	Scalable to large datasets; applicable in both cloud and offline setups
KNN, NB, ERT, RBF, MLP [11]	Simple models; comparative evaluation	Shallow learners; lack hierarchical feature extraction	Learns multiscale deep features; better generalization than shallow models
ML with PCA, KMC, SHAP [12]	Feature interpretability with SHAP; various feature selection strategies	Limited to static patterns; not adaptive to complex inputs	Learns important features implicitly; adaptive to complex decision surfaces
14 ML classifiers using PyCaret [13]	Broad coverage of algorithms; automated tuning	No deep learning integration; poor temporal insight	Learns both shallow and deep dependencies; does not require extensive manual tuning
Survey-based ML models on lifestyle indicators [14]	Real-world data collection; diverse ML application	Limited feature space; traditional models used	Adapts to lifestyle and clinical features simultaneously; robust under diverse conditions
CNN + Fuzzy classifier + RNN + MF-CSA [15]	Metaheuristic optimization; deep feature extraction	High complexity; large filter size causes info loss	Lightweight filters preserve details; efficiently models both features and sequences
KECA + SMOTE + multiple ML classifiers [16]	Reduces dimensions; balances minority class	SMOTE overfits; lacks representation learning	Natural handling of skewed data; learns optimal features without manual transformation
Ensemble learning with survey-based data [17]	Combines prediction strengths; collected in a clinical setting	Ensemble complexity; no deep temporal modeling	Reduces need for ensembling by internal feature fusion; time-aware predictions
BiGRU + attention-based FCNN [18]	Captures key parts of sequences; multivariate analysis	Training time is high; sensitive to noise	Efficient sequence learning with low latency; improved robustness to noise

**Table 2:** Mapping of identified limitations with the proposed solutions.

Identified Limitations / Research Gap	Proposed Solutions
L1: SMOTE for dataset balancing [16,17,20]. Does not consider the decision boundary and performs oversampling for all the minority class instances, which leads to overfitting.	S1: ADASYN uses adaptive learning to generate synthetic data points, and it shifts the decision boundary towards harder-to-learn samples.
L2: A VGG16-based model for diabetes classification [21]. A very high number of parameters. Slow training and high computational cost. Vanishing gradient problem. Lack of diversity in feature selection with 3×3 kernel for all the convolutional layers.	S2: InceptionNet performs diverse feature selection with the help of three different convolutions in InceptionNet. In the proposed TIPNet deep model, InceptionNet and LSTM can capture static and temporal complex features as well.
L3: A hybrid model where CNN is employed for the feature extraction [15]. CNN uses 11×11 filters in the first convolution layer to extract features; however, due to the large size of the filter, a lot of information is lost.	S3: Our TIPNet deep model can overcome this issue because LSTM can handle long-term dependencies, and InceptionNet uses different-sized filters at the same level to extract diverse features.
L4: A GRU-based classifier for diabetes prediction [15,19]. BiGRU was used for diabetes prediction [18]. GRU does not perform well when there are complex features to extract, and data presents long-term dependencies. BiGRU is a complex model with a high execution time and cannot learn long sequences.	S4: In the TIPNet ensemble model, InceptionNet addresses the problem of vanishing gradient, and LSTM handles the long-term dependencies and sequences with the help of cell state.
L5: Authors in [22] use DNN for the prediction of diabetes. Inability to handle complex patterns and prone to overfitting.	S5: The TIPNet deep model can overcome this issue because LSTM can handle complex patterns, and InceptionNet uses different-sized filters at the same level to extract diverse features.

**Table 3:** Summary of methodology used.

Method	Description	Key Features	Applications
Removing Duplicate Values	Identifies and removes duplicate records in the dataset to ensure data integrity.	Reduces overfitting, improves data quality, and ensures unique instances.	Preprocessing step in medical datasets to improve training reliability.
ADASYN	Adaptive synthetic oversampling technique to balance class distributions.	Focuses on harder-to-learn minority samples, reduces bias, and adjusts decision boundaries.	Balancing imbalanced datasets for medical diagnosis.
TIPNet	A blending ensemble model combining LSTM, InceptionNet, and MLP for accurate prediction.	Integrates sequential and static feature processing, high precision, and reduces false positives (FPs).	Medical diagnostics with heterogeneous datasets.
10-FCV	Statistical validation technique to evaluate model performance on different data splits.	Ensures robust and consistent metrics, reduces overfitting, and evaluates generalization.	Validating ML and DL models.
SHAP	Explainable AI technique that calculates feature contributions to model predictions.	Enhances interpretability, highlights influential features, and improves trust in AI systems.	Interpreting predictions in diabetes diagnosis models.
LIME	Local explanation method for understanding specific model predictions.	Provides instance-level insights, and identifies key factors influencing decisions.	Explaining individual predictions in healthcare applications.

**Table 4:** Features and their descriptions from the diabetes health indicators dataset.

Feature	Description	Possible Values
Diabetes	Indicator for the presence of diabetes.	0, 1
HighBP	Diagnosed with hypertension.	0, 1
HighChol	Presence of high cholesterol levels.	0, 1
CholCheck	cholesterol levels within the last five years.	0, 1
BMI	Individual’s body mass index.	12–98
Smoker	Current or past smoking habits.	0, 1
Stroke	History of stroke diagnosis.	0, 1
HeartDiseaseorAttack	Diagnosed with heart disease or heart attack.	0, 1
PhysActivity	Engaged in physical activities in the past month.	0, 1
Fruits	Regular consumption of fruits	0, 1
Veggies	Regular consumption of vegetables.	0, 1
HvyAlcoholConsump	High intake of alcoholic beverages.	0, 1
AnyHealthcare	Access to healthcare services.	0, 1
NoDocbcCost	Unable to consult a doctor due to financial constraints.	0, 1
GenHlth	Overall self-assessed health condition.	1–5
MentHlth	Number of days within past month when mental issues have occurred.	0–30
PhysHlth	Days spent experiencing physical health problems throughout past month.	0–30
DiffWalk	Having trouble using the stairs or walking.	0, 1
Sex	Biological sex.	0, 1
Age	Age group spanning from 18 to 80+ years.	1–13
Education	Educational attainment level.	1–6
Income	Household income range.	1–8

**Table 5:** Features and their descriptions from the DiaHealth dataset.

Feature	Description	Possible Values
age	Age of the patient in years	Continuous
gender	Biological sex of the patient	Male, Female
pulse_rate	Heart rate measured in beats per minute	Continuous
systolic_bp	Systolic blood pressure measured in mmHg	Continuous
diastolic_bp	Diastolic blood pressure measured in mmHg	Continuous
glucose	Blood glucose concentration (mg/dL)	Continuous
height	Height of the patient in centimeters	Continuous
weight	Weight of the patient in kilograms	Continuous
bmi	Body Mass Index calculated as weight/height^2^	Continuous
family_diabetes	Family history of diabetes	Yes, No
hypertensive	Whether the patient has hypertension	Yes, No
family_hypertension	Family history of hypertension	Yes, No
cardiovascular_disease	History of cardiovascular diseases	Yes, No
stroke	Whether the patient has had a stroke	Yes, No
diabetic	Diabetes status of the patient (target variable)	Yes, No

**Table 6:** Evaluation metrics and formulas.

Metric	Formula
Accuracy	$\frac{TP+TN}{TP+TN+FP+FN}$
Precision	$\frac{TP}{TP+FP}$
Recall	$\frac{TP}{TP+FN}$
F1-Score	$2\times \frac{Precision\times Recall}{Precision+Recall}$
AUC-ROC	$AUC=\int_{0}^{1}TPR\left(FPR\right)\;dFPR$

**Table 7:** Performance comparison of Temporal Inception Perceptron Network and baseline models for diabetes prediction.

Model	Accuracy	F1-Score	Recall	Precision	AUC	Exe Time (s)
HighwayNet	0.74	0.72	0.74	0.69	0.73	71
LeNet	0.75	0.73	0.79	0.69	0.76	127
TCN	0.75	0.76	0.80	0.73	0.75	298
LSTM	0.85	0.84	0.80	0.89	0.83	507
InceptionNet	0.85	0.86	0.88	0.83	0.84	196
MLP	0.76	0.77	0.81	0.73	0.79	103
TIPNet Deep Model	0.88	0.89	0.89	0.89	0.90	735

**Table 8:** Performance comparison of Temporal Inception Perceptron Network and baseline models for diabetes prediction for DiaHealth Dataset.

Model	Accuracy	F1-Score	Recall	Precision	AUC	Exe Time (s)
HighwayNet	0.74	0.79	0.79	0.65	0.90	5
LeNet	0.70	0.75	0.82	0.63	0.83	2
TCN	0.77	0.77	0.82	0.74	0.85	3
LSTM	0.75	0.75	0.77	0.73	0.83	16
InceptionNet	0.84	0.82	0.80	0.83	0.91	6
MLP	0.73	0.78	0.81	0.63	0.92	5
TIPNet Deep Model	0.87	0.86	0.88	0.84	0.93	29

**Table 9:** Accuracy insights for proposed and baseline models.

Model	Accuracy	Insights
HighwayNet	0.74	HighwayNets mitigate vanishing gradient issues by using gated mechanisms to learn feature hierarchies. However, they lack the capacity for sophisticated feature extraction and do not adapt well to datasets with both static and temporal features. Their relatively shallow architecture limits their ability to model complex medical patterns in the diabetes dataset.
LeNet	0.75	LeNet’s foundational convolutional architecture is effective for image-based tasks but falls short in handling mixed data types like medical indicators. The network’s simplicity and smaller number of layers lead to limited performance in extracting nuanced relationships between static features and sequential trends.
TCN	0.75	TCNs specialize in modeling sequential dependencies and excel in temporal data processing. However, their reliance on fixed receptive fields restricts their ability to capture fine-grained relationships in static features, which are equally critical for diabetes prediction. The lack of a mechanism to balance static and temporal feature processing limits their effectiveness.
LSTM	0.85	LSTM effectively captures temporal dependencies, making it suitable for sequential data like glucose trends. However, its inability to process static features directly limits its holistic understanding of the dataset.
InceptionNet	0.85	InceptionNet excels at multi-scale feature extraction, processing diverse patterns in static data. Its lack of temporal processing capability results in comparable accuracy to LSTM but restricts its performance on sequential tasks.
MLP	0.76	MLP struggles with sequential data as it lacks temporal feature learning mechanisms. Its accuracy reflects its reliance solely on static feature interactions, which are insufficient for complex temporal relationships.
TIPNet	0.88	TIPNet integrates temporal (LSTM) and static (InceptionNet) processing through a blending mechanism, achieving superior accuracy by leveraging complementary strengths. Its ensemble approach ensures balanced performance.

**Table 10:** Architectures of Temporal Inception Perceptron Network and baseline deep models for diabetes prediction.

Models	Architectures
LSTM (Base Model)	LSTM(Number of neurons=128, activation function=‘tanh’, return sequences= False)
	Dense layer(Number of neurons=1, activation function=‘sigmoid’)
InceptionNet (Base Model)	tower1=Conv1D(Number of neurons=16, filter=1, padding=’same’, activation function=‘tanh’)(X)
	tower2=Conv1D(Number of neurons=32, filter=1, padding=’same’, activation function=‘tanh’)(X)
	tower2=Conv1D(Number of neurons=32, filter=3, padding=’same’, activation function=‘tanh’)(tower2)
	tower3=Conv1D(Number of neurons=32, filter=1, padding=’same’, activation function=‘tanh’)(X)
	tower3=Conv1D(Number of neurons=32, filter=5, padding=’same’, activation function=‘tanh’)(tower3)
	tower4=MaxPooling1D(filter=3, strides=1, padding=’same’)(X)
	Output=Concatenate([tower1, tower2, tower3, tower4])
	Output=Flatten()(Output)
	Dense layer(Number of neurons=16, activation function=‘tanh’)(Output)
	Dropout(0.5)
	Dense layer(Number of neurons=1, activation function=‘sigmoid’)
MLP(Meta Model)	Dense layer(Number of neurons=256, activation function=‘ReLU’)
	Dense layer(Number of neurons=128, activation function=‘ReLU’)
	Dense layer(Number of neurons=64, activation function=‘ReLU’)
	Dense layer(Number of neurons=32, activation function=‘ReLU’)
	Dropout(0.2)
	Dense layer(Number of neurons=1, activation function=‘sigmoid’)

**Table 11:** Hyper-parameters utilized in Temporal Inception Perceptron Network and baseline deep models for diabetes prediction.

**Common Parameters:**	
Loss Function	Binary cross_entropy
Optimizer	Adam
Metrics	Accuracy
Epoch	5–15
Batch Size	32
**LSTM:**	
LSTM Layers	1
Activation Function in LSTM layer	tanh
Number of Neurons	128
Activation Function on Output Layer	Sigmoid
**InceptionNet:**	
Convolutional Layers	6
Convolutional Layers Filter Size	4: 1 × 1, 1: 5 × 5 and 1: 3 × 3
Number of Neurons in Convolutional Layers	16 for 1–32 for 5
Activation Function in Convolutional Layers	tanh
MaxPooling Layers	1 with filter size 3 and stride 1
Dense Layers	1 with 16 neurons and tanh activation function
Dropout Rate	0.5
Activation Function on Output Neuron	Sigmoid
**MLP:**	
Hidden Dense Layers	4
Activation Function in every Hidden layer	ReLU
Number of Neurons	32–256
Dropout Rate	0.2
Activation Function on Output Layer	Sigmoid

**Table 12:** Performance metrics of TIPNet with different random states using ADASYN.

TIPNet				
Metrics	Random State = 2	Random State = 4	Random State = 6	Random State = 8
Accuracy	0.88	0.85	0.85	0.84
F1-score	0.89	0.83	0.84	0.83
Recall	0.89	0.77	0.83	0.82
Precision	0.89	0.90	0.85	0.85
AUC	0.90	0.89	0.91	0.90
Exe time (s)	735	150	136	181

**Table 13:** 10-FCV results of Temporal Inception Perceptron Network for diabetes prediction.

**Metric**	**F1**	**F2**	**F3**	**F4**	**F5**	**F6**	**F7**	**F8**	**F9**	**F10**	**Average**
Accuracy	0.88	0.88	0.87	0.87	0.89	0.88	0.88	0.88	0.88	0.88	0.88
F1-Score	0.86	0.86	0.86	0.84	0.88	0.87	0.87	0.87	0.86	0.86	0.86
Recall	0.77	0.76	0.77	0.73	0.83	0.77	0.78	0.80	0.79	0.78	0.78
Precision	0.98	0.99	0.97	0.99	0.94	0.98	0.96	0.96	0.94	0.96	0.97

**Table 14:** Evaluation of TIPNet with 95% confidence intervals.

Metric	Mean	95% Confidence Interval
Accuracy	0.88	[0.87, 0.88]
F1-Score	0.86	[0.86, 0.87]
Recall	0.78	[0.76, 0.80]
Precision	0.97	[0.95, 0.98]

**Table 15:** Feature ablation for Temporal Inception Perceptron Network.

Removed Features	Accuracy	Removed Features	Accuracy
HighBP	0.828	HighChol	0.828
CholCheck	0.850	BMI	0.805
Smoker	0.849	Stroke	0.868
HeartDiseaseorAttack	0.846	PhysAct	0.840
Fruits	0.828	Veggies	0.844
HvyAlcoholConsump	0.819	AnyHealthcare	0.820
NoDocbsCost	0.856	MenHlth	0.847
DiffWalk	0.859	Sex	0.841
Age	0.848	Education	0.862
Income	0.832	PhysHlth	0.835
GenHlth	0.854	Base TIPNet	0.850

**Table 16:** Model ablation results for Temporal Inception Perceptron Network.

Modify/Remove Component	Accuracy	Modify/Remove Component	Accuracy
LSTM Layer 64 Units	0.835	LSTM Layer 96 Units	0.807
Inception module dense layer	0.790	Inception 3 × 3 convolutional layer	0.819
Inception 5 × 5 convolutional layer	0.829	MLP dropout	0.827
MLP dense layer 1	0.831	MLP dense layer 2	0.831
MLP dense layer 3	0.843	SGD instead Adam optimizer	0.685

**Table 17:** Performance comparison of models at different thresholds.

Metrics	Threshold > 0.3				Threshold > 0.7			
	LSTM	InceptionNet	MLP	TIPNet	LSTM	InceptionNet	MLP	TIPNet
Accuracy	0.78	0.81	0.80	0.81	0.81	0.84	0.78	0.84
F1-score	0.79	0.82	0.82	0.82	0.73	0.83	0.74	0.81
Recall	0.84	0.90	0.90	0.91	0.59	0.76	0.60	0.69
Precision	0.75	0.76	0.75	0.75	0.95	0.91	0.96	0.98
AUC	0.79	0.82	0.80	0.81	0.75	0.80	0.78	0.79
Exe Time (s)	433.90	88.56	45.44	47.82	349.75	86.99	46.37	49.28

## Data Availability

The data presented in this study are openly available in https://www.kaggle.com/datasets/alexteboul/diabetes-health-indicators-dataset, accessed on 12 July 2024.

## Abbreviations

The following abbreviations are used in this manuscript:

**Abbreviation**	**Full Form**
ADASYN	Adaptive Synthetic Oversampling
AI	Artificial Intelligence
BRFSS	Behavioral Risk Factor Surveillance System
CNN	Convolutional Neural Network
DL	Deep Learning
DNN	Dense Neural Network
FN	False Negative
FP	False Positive
LIME	Local Interpretable Model-Agnostic Explanations
LSTM	Long Short-Term Memory
ML	Machine Learning
MLP	Multilayer Perceptron
SHAP	SHapley Additive exPlanations
TIPNet	Temporal Inception Perceptron Network
TN	True Negative
TP	True Positive
**Symbol**	**Description**
$X_{static}$	Static features (e.g., age, BMI, blood pressure)
$X_{temporal}$	Temporal features (e.g., glucose levels, activity logs)
*Y*	Target labels (0 for non-diabetic, 1 for diabetic)
$\hat{Y}$	Predicted probabilities
$W_{h},U_{h},b_{h}$	Weights and biases for the temporal module
$W^{\left(l\right)},b^{\left(l\right)}$	Weights and biases for the *l*-th perceptron layer
$W_{c},b_{c}$	Weights and biases for the fusion layer
$W_{out},b_{out}$	Weights and biases for the output layer
$h_{t}$	Hidden state at time *t*
$H_{temporal}$	Temporal feature representation
$H_{static}$	Static feature representation
*z*	Concatenated and transformed feature representation
σ	Non-linear activation function (e.g., ReLU or Tanh)
sigmoid	Activation function for output probability
L(θ)	Binary cross-entropy loss
α	Learning rate for parameter updates
θ	Trainable parameters (weights and biases)
